# Supplementary material for: Evaluating the clinical effectiveness of the NHS Health Check programme: a prospective analysis in the Genetics and Vascular Health Check (GENVASC) study
Source: BMJ Open. 2023 May 30;13(5):e068025. doi: 10.1136/bmjopen-2022-068025 (PMC10230936; doi:10.1136/bmjopen-2022-068025)

SUPPLEMENTARY DATA

Clinical variables and measurements

BP was measured individually in GP practices using standardised oscillatory BP measuring devices after a minimum of 5-minute rest. Total Cholesterol (TC, mmol/l), High Density Lipoprotein-cholesterol (HDL-C, mmol/l) and serum creatinine (umol/l) were measured in accredited local laboratories following standard procedures. Non-HDL cholesterol (non-HDL-C, mmol/l) was calculated by subtracting value of HDL cholesterol from TC, Low Density Lipoprotein-cholesterol (LD-CL, mmol/l) was calculated using the Friedewald equation and estimated glomerular filtration rate (eGFR, ml/min/1.73xm^2) was calculated using the MDRD formula.

Townsend Deprivation Score

The Townsend Score is a composite measure of material deprivation of the population in UK. The Index was first introduced in 1987. The Townsend Score is published by the Office of National Statistics. Townsend score is calculated using four census variables for any given geographical area. These variables include: unemployment - defined as the percentage of economically active residents who are unemployed; non-car ownership measuring the number of households without a car; non-home ownership measuring the number of households renting and owning their accommodations; overcrowding – measuring whether the accommodation is suitable in size for the number of occupants. The Townsend Deprivation Scores were calculated using percentages of the weighted and normalised (where appropriate) four previously listed indicators. Townsend Deprivation Scores are usually split into quintiles to provide map visualisations of deprivation and to assess the spread of deprivation across areas.

https://statistics.ukdataservice.ac.uk/dataset/2011-uk-townsend-deprivation-scores

Debiec R, *et al. BMJ Open* 2023; 13:e068025. doi: 10.1136/bmjopen-2022-068025

Supplementary Table 1. Participants' baseline characteristics according to CVD risk levels at the initial Health Check.

	All participants n=27,888	CVD risk<br>Low QRISK2 <10% n=21,636 (78)	High QRISK2 10%-20% n=5,090 (18)	Very high QRISK2>20% n=1,162 (4)	Data Coverage (%)
**Demographic**					
Males, n (%)	12,322 (44)	8,206 (38)	3,118 (61)	998 (86)	100.0
Age, years	51 (44; 60)	48 (43; 54)	65 (60; 69)	69 (64; 72)	100.0
Ethnicity, n (%)					99.5
White	22,677 (81)	17,212 (80)	4,477 (88)	988 (85)	
Asian	3,686 (13)	3,030 (14)	499 (9.8)	157 (13)	
Black	535 (1.9)	501 (2.3)	33 (0.7)	1 (0.1)	
Other ethnicity	990 (3.6)	893 (4.1)	81 (1.6)	16 (1.4)	
Quintiles of deprivation, n (%)					97.6
1st quintile (least deprived)	8,332 (30.6)	6,436 (30.6)	1,587 (31.7)	309 (27.1)	
2nd quintile	5,867 (21.6)	4,509 (21.4)	. 1,126 (22.4)	232 (20.3)	
3rd quintile	4,429 (16.3)	3,417 (16.2)	832 (16.6)	180 (15.8)	
4th quintile	3,415 (12.5)	2,689 (12.8)	583 (11.6)	143 (12.5)	
5th quintile (most deprived)	5,167 (19.0)	4,003 (19.0)	887 (17.7)	277 (24.3)	
**Clinical**					
Smoking					82.6
Non-smoker	13,810 (60)	11,416 (64)	2,081 (49)	313 (31)	
Ex-smoker	5,415 (23)	3,738 (21)	1,304 (31)	373 (38)	
Current smoker	3,813 (17)	2,673 (15)	834 (20)	306 (31)	
Obesity					
BMI, kg/m^2	27.9 (5.1)	27.1 (5.1)	27.5 (5.0)	28.2 (5.1)	100.0
Abnormal body weight (BMI≥25, BMI>23for Asian subjects), n (%)	18,443 (66.1)	14,099 (65.2)	3,458 (67.9)	886 (76.2)	
Blood pressure					

Debiec R, *et al. BMJ Open* 2023; 13:e068025. doi: 10.1136/bmjopen-2022-068025

SBP, mmHg	127.6 (16.0)	124.9 (14.7)	135.6 (15.9)	143.6 (18.9)	100.0
DBP, mmHg	78.8 (10.0)	78.1 (9.7)	80.6 (10.4)	83.4 (11.7)	97.0
Elevated BP (SBP≥140mmHg and/or DBP≥90mmHg), n (%)	7,005 (25.1)	4,251 (19.7)	2,079 (40.8)	675 (58.1)	
Cholesterol					
TCh, mmol/l	5.29 (0.97)	5.22 (0.95)	5.53 (0.99)	5.53 (1.04)	81.5
Non-HDL, mmol/l	3.76 (0.96)	3.67 (0.94)	3.98 (0.96)	4.17 (1.00)	44.0
Elevated TCh (TCh≥5mmol/l), n (%)	14,140 (62.2)	10,449 (59.3)	3,016 (71.9)	675 (72.4)	
eGFR					
eGFR, ml/min/1.73m^2	90 (83, 90)	90 (85; 90)	90 (79; 90)	87 (78; 90)	73.5
Abnormal eGFR (eGFR<60 ml/min/1.73m^2), n (%)	174 (0.9)	94 (0.6)	60 (1.6)	20 (2.3)	

Results are mean (SD) or median (1st quartile; 3rd quartile) unless otherwise stated; Data coverage refers to the proportion of subjects with available data. **Abbreviations:** BMI-body mass index; SBP-systolic blood pressure; DBP-diastolic blood pressure; TCh-total cholesterol; Quintiles of deprivation were calculated using the Townsend Index of Deprivation 2019.[27] For the calculation of Qrisk2, Townsend index of deprivation was substituted with the average value of the index (i.e. “0”) in participants with missing data.

Supplementary Figure 1. Geographical distribution of recruitment to the GENVASC study

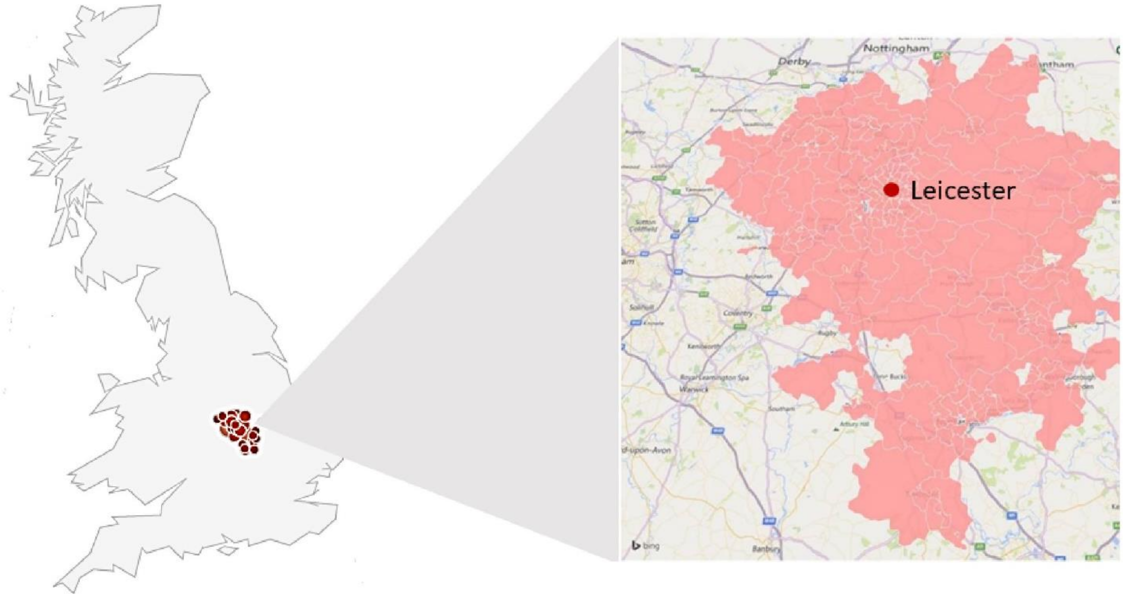

Supplement: Supplementary data [file bmjopen-2022-068025supp001.pdf]
