# Supplementary material for: Evaluating the clinical effectiveness of the NHS Health Check programme: a prospective analysis in the Genetics and Vascular Health Check (GENVASC) study
Source: BMJ Open. 2023 May 30;13(5):e068025. doi: 10.1136/bmjopen-2022-068025 (PMC10230936; doi:10.1136/bmjopen-2022-068025)
Supplement: Author's manuscript [file bmjopen-2022-068025.draft_revisions.pdf]

BMJ Open is committed to open peer review. As part of this commitment we make the peer review history of every article we publish publicly available.

When an article is published we post the peer reviewers' comments and the authors' responses online. We also post the versions of the paper that were used during peer review. These are the versions that the peer review comments apply to.

The versions of the paper that follow are the versions that were submitted during the peer review process. They are not the versions of record or the final published versions. They should not be cited or distributed as the published version of this manuscript.

BMJ Open is an open access journal and the full, final, typeset and author-corrected version of record of the manuscript is available on our site with no access controls, subscription charges or pay-per-view fees (http://bmjopen.bmj.com).

If you have any questions on BMJ Open's open peer review process please email info.bmjopen@bmj.com

BMJ Open

Clinical effectiveness of the NHS Health Check programme: Insights from the Genetics and Vascular Health Check (GENVASC) Study

Journal:	*BMJ Open*
Manuscript ID	bmjopen-2022-068025
Article Type:	Original research
Date Submitted by the Author:	14-Sep-2022

Complete List of Authors:	Debiec, Radoslaw; University of Leicester, Department of Cardiovascular Sciences and NIHR Cardiovascular Research Centre<br>Lawday, Daniel; University of Leicester, Department of Cardiovascular Sciences and NIHR Cardiovascular Research Centre<br>Bountziouka, Vasiliki; University of Leicester, Department of Cardiovascular Sciences and NIHR Cardiovascular Research Centre; University of the Aegean, Department of Food Science and Nutrition<br>Beeston, Emma; University of Leicester, Department of Cardiovascular Sciences and NIHR Cardiovascular Research Centre<br>Greengrass, Chris; University of Leicester, Department of Cardiovascular Sciences and NIHR Cardiovascular Research Centre<br>Bramley, Richard; University of Leicester, Department of Cardiovascular Sciences and NIHR Cardiovascular Research Centre<br>Sehmi, Sue; University of Leicester, Department of Cardiovascular Sciences and NIHR Cardiovascular Research Centre<br>Kharodia, Shireen; University of Leicester, Department of Cardiovascular Sciences and NIHR Cardiovascular Research Centre<br>Newton, Michelle; University of Leicester, Department of Cardiovascular Sciences and NIHR Cardiovascular Research Centre<br>Marshall, Andrea; University of Leicester, Department of Cardiovascular Sciences and NIHR Cardiovascular Research Centre<br>Krzeminski, Andre; Albany House Medical Centre<br>Zafar, Azhar; University of Leicester, Diabetes Research Centre; Diabetes and Cardiovascular Medicine General Practice Alliance Federation Research and Training Academy<br>Chahal, Anuj; South Leicestershire Medical Group<br>Heer, Amardeep; Lakeside Healthcare Research<br>Khunti, Kamlesh; University of Leicester, Diabetes Research Centre<br>Joshi, Nitin; Willowbrook Medical Centre<br>Lakhani, Mayur; University of Leicester, Department of Health Sciences<br>Farooqi, Azhar; NIHR Applied Research Collaboration<br>Patel, Riyaz; University College London, Institute of Cardiovascular Science<br>Samani, Nilesh; University of Leicester, Department of Cardiovascular Sciences and NIHR Cardiovascular Research Centre

Keywords:	PREVENTIVE MEDICINE, Health policy < HEALTH SERVICES ADMINISTRATION & MANAGEMENT, Vascular medicine < INTERNAL MEDICINE

SCHOLARONE™
Manuscripts

I, the Submitting Author has the right to grant and does grant on behalf of all authors of the Work (as defined in the below author licence), an exclusive licence and/or a non-exclusive licence for contributions from authors who are: i) UK Crown employees; ii) where BMJ has agreed a CC-BY licence shall apply, and/or iii) in accordance with the terms applicable for US Federal Government officers or employees acting as part of their official duties; on a worldwide, perpetual, irrevocable, royalty-free basis to BMJ Publishing Group Ltd ("BMJ") its licensees and where the relevant Journal is co-owned by BMJ to the co-owners of the Journal, to publish the Work in this journal and any other BMJ products and to exploit all rights, as set out in our licence.

The Submitting Author accepts and understands that any supply made under these terms is made by BMJ to the Submitting Author unless you are acting as an employee on behalf of your employer or a postgraduate student of an affiliated institution which is paying any applicable article publishing charge ("APC") for Open Access articles. Where the Submitting Author wishes to make the Work available on an Open Access basis (and intends to pay the relevant APC), the terms of reuse of such Open Access shall be governed by a Creative Commons licence – details of these licences and which Creative Commons licence will apply to this Work are set out in our licence referred to above.

Other than as permitted in any relevant BMJ Author's Self Archiving Policies, I confirm this Work has not been accepted for publication elsewhere, is not being considered for publication elsewhere and does not duplicate material already published. I confirm all authors consent to publication of this Work and authorise the granting of this licence.

For peer review only

Clinical effectiveness of the NHS Health Check programme: Insights from the Genetics and Vascular Health Check (GENVASC) Study

Running title: Clinical effectiveness of the NHS Health Check programme

Radoslaw Debiec[a], Daniel Lawday[a], Vasiliki Bountziouka[a,b], Emma Beeston[a], Chris Greengrass[a], Richard Bramley[a], Sue Sehmi[a], Shireen Kharodia[a], Michelle Newton[a], Andrea Marshall[a], Andre Krzeminski[c], Azhar Zafar[d,e], Anuj Chahal[f], Amardeep Heer[g], Kamlesh Khunti[d], Nitin Joshi[h], Mayur Lakhani[i], Azhar Farooqi[i], Riyaz Patel[j], Nilesh J Samani[a]

Affiliations of authors:

a) Department of Cardiovascular Sciences, University of Leicester and NIHR Cardiovascular Research Centre, Glenfield Hospital, Leicester, UK

b) Department of Food Science and Nutrition, University of the Aegean, Lemnos, Greece

c) Albany House Medical Centre, Wellingborough, UK

d) Diabetes Research Centre, University of Leicester, UK

e) Diabetes and Cardiovascular Medicine General Practice Alliance Federation Research and Training Academy, UK

f) South Leicestershire Medical Group, Kibworth Beauchamp, UK

g) Lakeside Healthcare, Corby, UK

h) Willowbrook Medical Centre, Leicester, UK

i) Department of Health Sciences, University of Leicester, UK

j) Institute of Cardiovascular Science, University College London, London, UK

Address for correspondence:

Dr Radoslaw Debiec,

Department of Cardiovascular Sciences and NIHR Leicester Biomedical Research Centre,

Glenfield Hospital,

Groby Road, LE39QP

rmd24@leicester.ac.uk

For peer review only - http://bmjopen.bmj.com/site/about/guidelines.xhtml

Tel: 01162044789

Disclaimers: KK was a national advisor for the NHS Health Checks Programme

Word Count: 3988

Sources of support

The GENVASC study is funded by the NIHR Leicester Biomedical Research Centre (Grant No. BRC-1215-20010)

For peer review only

For peer review only - http://bmjopen.bmj.com/site/about/guidelines.xhtml

Abstract

Objective

The aim of the study was to assess the clinical effectiveness of the national Cardiovascular Disease (CVD) prevention program - NHS Health Check (NHSHC). Specifically the study assessed NHSHC attributed detection of CVD and risk factors, prescription of medications, changes in values of individual risk factors and frequency of follow up.

Methods: The analysis comprised 27,888 individuals undergoing NHS health checks across 147 General Practices (GP) in England between 2010 – 2018 with a minimum of 18 months of follow up.

Results: At recruitment, 18% of participants had high CVD risk (10-20% 10-year risk) and 4% very high CVD risk (>20% 10-year risk). New diagnoses of hypertension were made in 2.3%, hypercholesterolaemia in 0.25%, diabetes mellitus in 0.9 %. The check led to new prescription of stains in 5.4% and anti-hypertensive medications in 5.4% of participants and reduction of total cholesterol (by 0.38 mmol/l (-0.34;-0.41, p<0.001) and 1.71 mmol/l (-1.48;-1.94, p<0.001) in patients with initial cholesterol >5mmol/l and >7.5mmol/l, respectively and systolic blood pressure by 2.9 mmHg (-2.3;-3.7, p<0.001), 15.7 mmHg (-14.1;-17.5, p<0.001) and 33.4 mmHg (-29.4;-37.7, p<0.001), respectively in patients with grade one, two and three hypertension, respectively). About 1 out of 3 patients with high CVD risk had no record of follow-up or treatment.

Conclusions: Patients with increased CVD risk and attending follow-up receive effective clinical interventions. However, one third of high CVD risk patients were not followed up and therefore did not receive any treatment. Our study highlights novel findings which may influence implementation of strategies for improving effectiveness of the programme

Key words:

NHS health check, primary prevention, cardiovascular, clinical effectiveness

Strengths and limitations of the study

For peer review only - http://bmjopen.bmj.com/site/about/guidelines.xhtml

- Prospective observation of large cohort of patients from 147 GP Practices undergoing NHSHC
- Purposefully built, comprehensive database
- Possible selection bias – individuals volunteering to take part in the study might represent more “proactive” subgroup of NHSHC attendees
- Larger than national average proportion of non-white ethnicities representing population structure of the region

For peer review only

For peer review only - http://bmjopen.bmj.com/site/about/guidelines.xhtml

Introduction

Cardiovascular disease (CVD) is a leading cause of premature morbidity and mortality in England.[1, 2] To address this, in 2009 the UK government introduced first in the world rolling national CVD prevention programme, delivered by the National Health Service (NHS). Now known as the NHS Health Check (NHSHC), the programme aims to identify and treat the main risk factors driving CVD, among people aged 40-74 years who were otherwise well.[3] It was estimated through economic modelling that NHSHC could prevent over 1600 myocardial infarctions and strokes and 650 premature CVD deaths annually.[4]

The absence of randomised clinical trials demonstrating the effectiveness of such a programme, has continued to raise debate about its value.[5, 6] Instead, several studies sought to assess the effectiveness of the NHSHC using various observational datasets, but often with conflicting results, casting further doubt about the ability of the programme to achieve the stated goals.[7-11]

The Genetics and Vascular Health Check study (GENVASC) was instigated in 2012 to assess the value of adding a polygenic risk score (PRS) for CVD to the NHSHC for prediction of cardiovascular events. The study recruited patients attending for the NHSHC in 147 General Practices in two counties in England (Leicestershire and Northamptonshire) and collected comprehensive clinical data comprising the NHSHC as well as subsequent follow-up visits. This dataset now permits evaluation of the impact of NHSHC in more detail than previously reported. In particular, the dataset allows for detailed analysis of the sequelae of detecting a risk factor, prescription of pharmacotherapy, clinical follow-up and their correlation with observed reduction in the risk factor. Specifically, we were able to fully characterise the subpopulation of individuals, who attended the initial NHSHC and were identified to have high and very high cardiovascular risk as well as individuals with gross elevations of individual risk factors. The main aim of our study was to assess effectiveness of clinical interventions and identify factors that may influence clinical and cost effectiveness of the NHSHC.

Methods

<u>NHSHC</u>

For peer review only - http://bmjopen.bmj.com/site/about/guidelines.xhtml

The NHSHC is performed during a dedicated primary care visit and involves structured assessment of CVD risk.[12] Adults aged 40-74 years, without pre-existing CVD, diabetes mellitus, hypertension (HTN), hypercholesterolaemia and not taking statins are invited to attend the programme. The 10-year risk of developing CVD is calculated using QRISK2 score.[13] The attendees receive consultation regarding their CVD risk and individual risk factors: body mass index (BMI), tobacco smoking, blood pressure (BP), cholesterol level, alcohol intake, physical activity and risk of diabetes. Patients found to have increased overall CVD risk (QRISK2 score≥10%) or elevated individual risk factor values are given lifestyle modification advice, pharmacological treatment[14] and/or are referred to specialist services. Subjects with QRISK2<10% are invited for re-assessment every 5 years.

GENVASC study

GENVASC is a large observational study run in conjunction with Clinical Commissioning Groups and Primary Care practices in UK and coordinated by the NIHR Leicester Biomedical Research Centre. The GENVASC study recruited multi ethnic individuals aged 40-74 years, attending the NHSHC at any of the 147 participating General Practitioners' (GP) practices in Leicestershire and Northamptonshire, UK. **(Supplementary Figure 1)** The main aim of the study is to evaluate the additional clinical value of a PRS on top of traditional risk scores in prediction of subsequent CVD events.[15] Participants of the GENVASC are followed up using primary care databases. The collected information includes clinical diagnoses, laboratory and imaging tests, hospital admissions, and referrals to external services as well as medicinal prescriptions.

Study Participants

Our study sample comprised of 27,888 participants of the GENVASC study recruited between 17/06/2010 and 04/09/2018.This time limits were set to allow for 18 months of follow-up after the initial NHSHC.[15] Based on an estimate from Patel et al.[16] about the annual attendance to NHSHSC in Leicestershire and Northamptonshire, the population used for current analysis constituted around 10% of all NHSHC attendees in these two counties. All participants gave their consent to access their medical records during the duration of the study.

For peer review only - http://bmjopen.bmj.com/site/about/guidelines.xhtml

Clinical Variables and definitions

For details regarding methods of assessment and extraction of clinical and laboratory variables see Supplementary Material. Overall, 10-year CVD risk was defined from QRISK2 score and categorised as low (<10%), high (10-20%) and very high (>20%).[13] Blood pressure (BP) was defined as normal if systolic blood pressure (SBP) <140mmHg and/or diastolic blood pressure (DBP) <90mmHg; grade one hypertension (HTN) if SBP was 140-159mmHg and/or DBP 90-99mmHg, grade two HTN if SBP was 160-179mmHg and/or DBP 100-109mmHg, grade three HTN if SBP was≥180mmHg and/or DBP≥110mmHg.[17] Total cholesterol (TCh) was defined as normal for values <5.0mmol/l, high for TCh values 5.0-7.49mmol/l and very high for TCh ≥7.5mmol/l.[18] Non-high density lipoprotein (non-HDL) was defined as normal if values were <3.8mmol/l and high for values ≥3.8mmol/l.[18] Kidney function was considered normal if eGFR≥60ml/min/1.73xm²; chronic kidney disease (CKD) was diagnosed if eGFR<60ml/min/1.73xm².[19] Body weight was assessed using body mass index (BMI) and considered as healthy body weight if BMI <25kg/m^2 (<23 kg/m^2 for South Asians), overweight for BMI from 25 to 29.9kg/m^2 (23 to 27.4 kg/m^2 for South Asians) and obese if BMI ≥30 kg/m^2 (>27.5 for South Asians).

Baseline clinical observations were defined as measurements taken during the NHSHC or the nearest measurement taken around the date of NHSHC but within 30 days from the date of the check.

Any new diagnoses of CVD risk factors made from the date of NHSHC to 12 weeks after were attributed to the NHSHC visit. This 12-week period, although arbitrary, was considered appropriate for making clinical diagnosis and starting any treatment and supported by the bar plot of distribution of weekly diagnoses post NHSHC. **(Figure 1)**

Outcomes of interest

The assessment of the impact of the NHSHC was performed using the following measures:

- risk factor detection - absolute number and proportion of subjects diagnosed with an abnormal values of BP, TCh, eGFR, BMI or having a clinical diagnosis of HTN, diabetes, hypercholesterolaemia, CKD or atrial fibrillation

For peer review only - http://bmjopen.bmj.com/site/about/guidelines.xhtml

- medication prescription - absolute number and proportion of subjects started on a given therapy
- frequency of follow-up - number of clinical visits/reviews within the specified periods
- change in values of individual risk factors - difference between the value of a given clinical characteristic recorded during the NHSHC and first value of this characteristic recorded after 12 months from the NHSHC visit

Data Extraction

Data about participants were extracted from the GP information technology systems. The Primary care databases (EMIS and SystmOne) were used to obtain participants' socio-demographic information (age, sex, and ethnicity), anthropometric measurements (height, weight), information about health-related behaviour (tobacco smoking), clinical diagnoses, blood results and prescribed medicines. Relevant data were extracted using NHS Numbers, SNOMED CT/ DM&D and Clinical Terms Version 3 (CTV3) (formerly known as the Read codes).

Statistical analysis

Categorical variables (sex, ethnicity, deprivation based on quintiles of Townsend 2011 index (for further information on Townsend index see Supplementary Data – Townsend Deprivation Score), smoking status, BP category, TCh category, renal function category, overweight/ obesity, QRISK2 category) are shown as frequencies (relative frequency) whilst continuous data are shown as means and standard deviation (SD) if normally distributed (SBP, DBP, TCh), or median (interquartile range (IQR)) otherwise (age, eGFR). Normality was assessed using graphical methods (i.e. histograms, P-P and Q-Q plots).

Group comparisons were performed using a chi-square test for categorical variables and the one-way ANOVA for the normally distributed continuous variables or the Kruskall-Wallis for the non-normally distributed ones. The change in the levels of individual risk factors was calculated as the difference between the initial NHSHC measurement and the measurement during follow-up, after a period of twelve months. To assess the statistical

For peer review only - http://bmjopen.bmj.com/site/about/guidelines.xhtml

significance of that comparison, the paired t-test for the continuous and the McNemar test for the nominal data were used.

Subjects with no repeated values recorded for the measured variables from after the initial NHSHC visit to the total time of follow-up observation of 18 months were considered as not-followed up. The comparisons between individuals without follow up and those that were followed up were performed with the independent t-test and Pearson's chi-square test for the continuous and nominal data respectively. A logistic regression model was used to assess whether socio-demographic (age, sex, deprivation, ethnicity) and clinical factors (SBP, DBP, TCh, BMI, smoking) were associated with the absence of follow-up. Significance level was set to 0.05, all tests were two-sided and Stata v16.0 was used for all the calculations.[20]

Patient and public involvement:

GENVASC was developed in collaboration with the NIHR Leicester Biomedical Research Centre Cardiovascular Public Involvement Group. The research plan and all public facing materials were reviewed and approved prior to ethics submission. The group were updated biannually on the study. This was later extended to our tissue public involvement group (The Exceed Group).

Results

Key characteristics of the study cohort

Overall 27,888 GENVASC participants were included in the current analysis. The median age of included participants was 51 years, 44% (n=12,322) were men, 81% (n=22,677) were of White ethnicity and 13% (n=3,686) were South Asians. (**Supplementary Table 1**) The GENVASC population was, in general younger, had a higher proportion of Asians amongst the ethnic minorities, and had more participants from the extremes of the Townsend deprivation index (quintiles 1 and 5), compared to the NHSHC attendees across England or the general adult population aged between 40-74 years, albeit the differences were small. (**Table 1**)

For peer review only - http://bmjopen.bmj.com/site/about/guidelines.xhtml

Table 1. Comparison of GENVASC study participants included in the current analysis to the general population of England and large population of NHSHC attendees.

	ONS data n-22,805,612	NHSHC data* n=5,102,734	GENVASC study n=27,888	Missingness (%)
**Demographic**				
Males, n (%)	11,200,690 (49)	2 311 604 (45)	12,327 (44)	-
Age bands (years)				-
40-49	7,525,905 (33)	1,951,264 (38)	12,256 (43)	
50-59	7,089,322(31)	1,742,003 (34)	8,495 (30)	
60-74	8,190,476 (36)	1,409,491 (28)	7,137 (27)	
Ethnicity, n (%)				
White	20,383,677 (89)	4,067864 (80)	22,677 (81)	
Asian	1,341,581 (6)	368,145 (7)	3,686 (13)	
Black	585,756 (3)	148,160 (6)	535 (1.9)	
Other ethnicity	494,599 (2)	142,621 (2.8)	990 (3.6)	
Quintiles of deprivation, n (%)				43.5
1st quintile (least deprived)	4,996,212 (22)	1,129,607 (22)	4,757 (30)	
2nd quintile	4,901,834 (21)	1,094,925 (21)	3,302 (21)	
3rd quintile	4,707,382 (21)	1,027,096 (20)	2,523 (16)	
4th quintile	4,286,645 (19)	954,656 (19)	2,070 (13)	
5th quintile (most deprived)	3,913,539 (17)	893,194 (18)	3,390 (21)	

ONS - Office for National Statistics, NHSHC – NHS Health Check, GENVASC - The Genetics and Vascular Health Check study,

* Patel R, Barnard S, Thompson K, Lagord C, et al. Evaluation of the uptake and delivery of the NHS Health Check programme in England, using primary care data from 9.5 million people: a cross-sectional study. BMJ Open 2020;**10**(11):e042963.

Cardiovascular risk groups

At recruitment, 18% (n=5,090) of participants were classified to have high CVD risk and 4% (n=1,162) to have very high CVD risk. **(Supplementary Table 1).** Patients in the high

For peer review only - http://bmjopen.bmj.com/site/about/guidelines.xhtml

and very high CVD risk groups were on average older than patients in the low CVD risk groups (median age 65, 69 and 48 years, respectively), were more like to be male, be of White ethnic origin and current smokers. **(Supplementary Table 1)**

Diagnoses and risk factor detection

The majority of new clinical diagnoses of HTN, hypercholesterolaemia, diabetes mellitus and CKD occurred within the first 12 weeks following attendance to the NHSHC. **(Figure 1)** There was also a slight increase in the rate of new diagnoses of HTN, hypercholesterolaemia and diabetes between 12 weeks and six months post NHSHC. (**Figure 1**) After that period the rate of new diagnoses plateaued and remained stable through the study. (**Figure 1**) In contrast to HTN, hypercholesterolaemia and diabetes, the rate of new diagnoses of atrial fibrillation was similar through the study duration.

In the period from NHSHC to 12 weeks after, 2.3% (n=628) individuals received a coded diagnosis of HTN. There was a discrepancy between coded diagnoses of HTN and abnormal blood pressure measurements or antihypertensive medicine prescription. In the period from NHSHC to 12 weeks after, at least one abnormal BP measurements (SBP≥140mmHg and/or DBP≥90mmHg) was recorded for 27% (7,516) individuals. In the same period antihypertensive medications were prescribed to 5.4% (1,510) of individuals.

In the period from NHSHC till 12 weeks after, 0.25% (n=70) individuals received a coded diagnoses of hypercholesterolaemia. However, in the same period 58.7% (16,379) and 2% (547) individuals were found to have TCh ≥5mmol/l and TCh ≥7.5mmol/l, respectively and 5.4% (1,514/27,888) of all individuals were prescribed statins.

In the period form NHSC up to 12 weeks, 0.9% (n=248) patients were diagnosed with diabetes mellitus. Within the first 12 weeks post NHSHC coded diagnoses of obesity and CKD were recorded for 0.5% (n=144) and 0.07% (n=19) patients, respectively. However, within the same period values of BMI meeting criteria for obesity (≥30 kg/m^2 and ≥27.5 kg/m^2 for Asian subjects) and eGFR (<60 ml/min/1.73m2) were recorded for 26.2% (n=7,315) and 0.9% (n=174) patients.

Medical Prescriptions and clinical interventions

For peer review only - http://bmjopen.bmj.com/site/about/guidelines.xhtml

Within the first 12 weeks following the NHSHC, statins were prescribed to 5.4% (1,514/27,888) of all attendees and the proportion gradually increased to 7.1 % (1,984/27,888) by the end of the study follow-up period at 18 months. These proportions were correlated with the allocated CVD risk group at the NHSHC. Within the first 12 weeks following the NHSHC, statins were prescribed to 1.8%, 14.2% and 34.2% of individuals in the low, high and very high CVD risk groups respectively, reaching 2.8%, 18% and 39.2% by the end of the study duration at 18 months. The median time from the NHSHC to statin prescription was 29 weeks for the low CVD risk group, but reduced to seven and four weeks for the high and very high CVD risk groups. Within the first 12 weeks following the NHSHC, 5.3% of participants with high TCh and 24.5% of participants with very high TCh were started on statins, reaching 7.4% and 33%, respectively by the end of the study.

Following the NHSHC visit, 5.4% (1,510/27,888) of all attendees were started on antihypertensive medications within the first twelve weeks, which increased to 8.4% (2,337/27,888) by the end of study duration at 18 months. When categorised according to the initial CVD risk within the first 12 weeks following the NHSHC, antihypertensive medications were prescribed to 3.6%, 10.0% and 21.2% of individuals in the low, high and very high CVD risk groups respectively, reaching 6.4%, 14.8% and 26.2% respectively by the end of the study duration at 18 months.

Within the first 12 weeks from the NHSHC visit, of participants meeting criteria of grade one, grade two and grade three HTN, antihypertensive medications were prescribed to 9.7%, 26.7% and 61.2% of individuals, respectively. These proportions increased to 15.4%, 36.9% and 63.1% by the end of the follow-up at 18 months, respectively. At the end of the study 4.5%, 13.5% and 28.8% of GENVASC participants, meeting criteria for grade one, grade two and grade three HTN, respectively were prescribed more than one group of antihypertensive medications. The time from the NHSHC to onset of antihypertensive medication showed big discrepancies, however there was a strong trend for shorter time to pharmacological treatment with higher category of HTN. In particular, median

For peer review only - http://bmjopen.bmj.com/site/about/guidelines.xhtml

time from NHSHC to first prescription was reduced from 23 weeks for patients with grade one HTN, to 12 weeks for those with grade two HTN and 6 weeks for those with grade three HTN.

Frequency of follow-up

The frequency of follow-up visits were mainly dependent on the abnormalities detected during the NHSHC. Patients, with grade one, two and three hypertension during NHSHC visit had a median of 4 (95% CI 2-9), 4 (95% CI 2-11), and 6 (95% CI 2-12) measurements of BP, respectively, within 18 months of the study duration. In the whole length of 18 months of follow-up at least a single repeated measurement of BP was performed in 41% of patients with normal, 58% with grade one, 81% with grade two and 88.5% of patients with grade three HTN, respectively.

Similarly, within the duration of the study, repeated measurements of TCh were performed in 20%, 28% and 57% of patients with normal, high and very high TCh, respectively.

In contrast to the repeated measurements of BP and TCh repeated measurements of body weight were done less frequently. At least a single repeated weight measurement was recorded for 1,910 (17%) of overweight and 1,798 (25%) of obese participants in the follow-up period. Altogether, only around 1 in 4 of all subjects had a record of repeated weight measurements between the initial NHSHC visit and 18 months post.

Change in the levels of CVD risk factors

The magnitude of changes in the CVD risk factors during the follow-up period were correlated to the attendant's initial CVD risk group, allocated during the NHSHC visit.

For those in the low CVD risk category there was on average an increase in the SBP by 3.6mmHg and DBP by 1.2mmHg in those in whom measurements were repeated (n=6,785). Similarly, during the period of follow-up there was a reduction in mean eGFR by on average 5 ml/min/1.73m² in this group. **(Table 2**) In contrast to BP and eGFR, there was a minor reduction in the average levels of TCh by 0.14mmol/l. **(Table 2**)

For peer review only - http://bmjopen.bmj.com/site/about/guidelines.xhtml

Table 2. Comparison of clinical characteristic of participants between NHSHC and follow-up.

Parameters	NHSHC	Follow-up (12-18 months)	Delta (FU-HC) (95% CI)	P
**Low CVD risk**				
Obesity, n=3,747				
Body mass index, kg/m²	28.0 (5.9)	28.0 (5.9)	0.006 (-0.07; 0.05)	0.83
Blood pressure, n=6,785				
Systolic, mmHg	125.5 (15.6)	129.1 (16.3)	3.6 (3.1;3.9)	<0.001
Diastolic, mmHg	78.7 (10.2)	79.9 (10.4)	1.2 (1.0; 1.6)	<0.001
Cholesterol, n=3,233				
Total cholesterol, mmol/l	5.4 (1.0)	5.3 (0.98)	-0.14 (-0.16; -0.10)	<0.001
Non-HDL-cholesterol, n=1,339				
Non-HDL-cholesterol, mmol/l	3.9 (1.0)	3.7 (0.98)	-0.12 (-0.16; -0.08)	<0.001
eGFR, n=2,551				
eGFR, ml/min/1.73m²	86 (85; 87)	81 (75; 86)	-5 (-5.6; -4.3)	<0.0001
**High CVD risk**				
Obesity, n=1214				
Body mass index, kg/m²	27.6 (5.4)	27.6 (5.7)	-0.08 (-0.20; -0.06)	0.27
Blood pressure, n=2046				
Systolic blood pressure, mmHg	136.7 (16.5)	136.4 (16.6)	-0.3 (-1.1; 0.53)	0.5
Diastolic blood pressure, mmHg	81.1 (10.8)	80.2 (10.2)	-0.9 (-1.4; -0.40)	<0.001
Cholesterol, n=1,339				
Total cholesterol, mmol/l	5.6 (1.1)	5.1 (1.1)	-0.45 (-0.51; -0.39)	<0.001
Non-HDL-cholesterol, n=583				
Non HDL-cholesterol, mmol/l	4.0 (1.0)	3.6 (1.1)	-0.47 (-0.55; -0.39)	<0.001
eGFR, n=1,129				
eGFR, ml/min/1.73m²	82 (78; 81)	79 (78; 80)	-3 (-4.4; -1.6)	<0.001
**Very high CVD risk**				
Obesity, n=354				
Body mass index, kg/m²	28.7 (6.1)	28.4 (6.0)	-0.3 (-0.52; -0.11)	0.002
Blood pressure, n=571				
Systolic blood pressure, mmHg	146.2 (20.8)	138.7 (16.3)	-7.5 (-9.4; -5.7)	<0.001
Diastolic blood pressure, mmHg	84.8 (12.5)	79.8 (10.4)	-5 (-6.0; -3.9)	<0.001
Cholesterol, n=419				
Total cholesterol, mmol/l	5.6 (1.1)	4.8 (1.2)	-0.79 (-0.91; -0.69)	<0.001
Non-HDL-cholesterol, n=174				
Non HDL-cholesterol, mmol/l	4.1 (1.0)	3.4 (1.1)	-0.68 (-0.83; -0.53)	<0.001
eGFR, n=303				
eGFR, ml/min/1.73m²	81 (79; 83)	78 (76; 80)	-3 (-5.0; -0.8)	0.006

Results are shown as mean (SD) unless otherwise indicated. Differences in mean values or proportions between the follow-up and the NHSHC, along with the relevant 95% Confidence intervals, are also shown. P-values derived from the paired t-test for the continuous variables and the McNemar test for the nominal paired variables.

For peer review only

For peer review only - http://bmjopen.bmj.com/site/about/guidelines.xhtml

Participants in the high CVD risk group achieved on average reduction of 0.9mmHg - in DBP. (**Table 2**) There were also clinically significant reductions in total and non-HDL cholesterol by 0.45mmol/l and 0.47mmol/l, respectively. (**Table 2**). There was no significant change in the BMI at follow up.

For participants in the very high CVD risk group, the average reductions in the SBP and DBP levels were 7.5mmHg and 5mmHg respectively. **(Table 2)** The reductions in TCh and non-HDL levels were 0.79mmol/l and 0.68mmol/l, respectively. **(Table 2)** During the period of follow-up eGFR was reduced by on average 3ml/min/1.73m^2 and BMI was reduced by on average -0.3kg/m^2. **(Table 2)**

Analogous to the gradient of CVD risk, the magnitude of changes in the individual risk factors levels were also strongly associated with the severity of abnormality observed for that risk factor during the NHSHC. Overall, individuals with BP above 140/90 mmHg, as measured during the NHSHC, achieved an average reduction of 7mmHg of SBP and 5mmHg of DBP after 12 months from assessment. For grade one, grade two and grade three HTN patients, SBP fell by 3, 16 and 33 mmHg, respectively. (**Figure 2**) A similar pattern was observed for the TCh levels, with an average reduction of 0.38mmol/l and 1.71mmol/l, respectively, for patients with high and very high TCh. (**Figure 2**) This trend was not observed for body weight. In particular, there was a small difference in the magnitude of reduction of BMI over the follow-up period with overweight patients losing approximately 0.1kg/m^2 and obese patients losing on average 0.3 kg/m^2.(**Figure 2**)

<u>Smoking cessation</u>

During the NHSHC, 16% (3,813/23,623; 19% males, 14% females) were recorded as current smokers. Of the 3,813 current smokers, 56% (2,133; 53% males, 47% females) had a record of smoking cessation advice documented during or within 30 days of the NHSHC. This proportion increased to 60% (2,275) by the end of the study, whilst 14% of subjects had multiple records of advice for smoking cessation. By the end of the study 451(12%) participants quit smoking, according to the GP records.

For peer review only - http://bmjopen.bmj.com/site/about/guidelines.xhtml

Individuals with no record of follow-up

The demographic and clinical characteristics of high and very high CVD risk patients with unrecorded follow-up are presented in **Table 3**. Approximately 1 in 3 patients with high or very high CVD risk had no recorded evidence of a clinical follow-up in the 18 month period following the NHSHC. Lack of clinical follow-up was associated with White ethnic background. (**Table 3**) Participants without evidence of follow-up had, at the time of the NHSHC, on average lower values of BMI, SBP and DBP than individuals who had record of follow-up, albeit the differences were small. (**Table 3**) Results from the logistic regression analysis showed that non-white ethnicity (OR (95% CI): (1.79 (1.33; 2.42), p=0.001), younger age (-1.02 (-1.001; -1.04), p=0.04), increased SBP (1.02 (1.01; 1.03), p<0.001) and increased BMI (1.03 (1.01; 1.05), p=0.001), were independently associated with presence of follow-up record. 5.7% of participants, with high or very high CVD risk and no evidence for attending clinical follow-up were prescribed statins and 2% antihypertensive medications.

For peer review only - http://bmjopen.bmj.com/site/about/guidelines.xhtml

Table 3. Demographic and clinical characteristics of participants with high and very high CVD risk who did not have record of follow-up.

	Follow-up		Delta (N-Y)	P
	Yes	No (lost)	(95% CI)	
**High &very high CVD risk**	4,217	2,035		
Demographic				
Male sex, n (%)	2,738 (64.9)	1,378 (67.7)	2.8 (0.3; 5.2)	0.0290
White ethnicity n (%)	3,615 (85.7)	1,850 (90.9)	5,2 (3.5; 6.8)	<0.0001
Age, years (Median, CI)	64.2 (63.9-64.4)	64.6 (64.4-64.9)	0.47 (0.11; 0.83)	0.0104
Quintiles of deprivation, n (%)				
1st quintile (least deprived)	679 (26.8)	299 (27.9)	4.0 (1.7; 6.3)	0.0005
2nd quintile	501 (19.8)	216 (20.1)	0.3 (-1.8; 2.4)	0.7807
3rd quintile	419 (16.5)	165 (15.4)	-1.1 (-3.0; 0.8)	0.2680
4th quintile	319 (12.6)	153 (14.3)	-1.7 (-3.4; 0.1)	0.0674
5th quintile (most deprived)	617 (24.3)	239 (22.3)	-2.0 (-4.2; 0.2)	0.0812
Clinical				
Current smokers, n (%)	781 (18.5)	359 (17.6)	-0.9 (-0.03; 0.11)	0.3876
Ex-smokers, n (%)	1,176 (27.9)	501 (24.6)	-3.3 (-5.6; -0.9))	0.0058
BMI, kg/m2	27.9 (5.2)	26.9 (4.7)	-1.0 (-1.0; -0.66)	<0.0001
Systolic blood pressure, mmHg	139.3 (17.8)	132.6 (13.5)	-6.7 (-7.0; -5.9)	<0.0001
Diastolic blood pressure, mmHg	82.3 (11.2)	78.7 (8.9)	-3.6 (-4.0; 3.1)	<0.0001
Antihypertensive medications, n (%)	1,012 (24.0)	44 (2.2)	-21.8 (-23.2; -20)	<0.0001
Total cholesterol, mmol/l	5.6 (1.1)	5.5 (1.0)	-0.1 (-0.1; -0.02)	0.0079
Non-HDL cholesterol, mmol/l	4.0 (1.0)	3.9 (0.9)	-0.1 (-0.1, 0.01)	0.0622
Statins, n (%)	1,257 (29.8)	116 (5.7)	-24.1(-25.8-; -22.4)	<0.0001

Results are mean (SD) unless otherwise stated.

For peer review only - http://bmjopen.bmj.com/site/about/guidelines.xhtml

Discussion

We present results evaluating the clinical effectiveness of the NHSHC in identifying, treating and monitoring individuals at high CVD risk. Our study highlights novel findings which may influence implementation of strategies for improving effectiveness of the programme.

A key finding from our study is the characterisation of high and very high CVD risk patients, who had attended the NHSHC, but have not had any record of clinical follow-up 18 months after the first visit, and have not received any treatment. This is a considerably large sub-population of patients (33%) and our study showed that this group has specific social, ethnic and clinical characteristics. These patients represent a missed opportunity for CVD prevention. It is also the group of patients, where preventive interventions are likely to bring the largest clinical benefits and be most cost effective. Similar observations were made for patients with very abnormal values of isolated risk factors - such as blood pressure and cholesterol. Only around 29% of patients with BP reading meeting criteria for grade three HTN had been prescribed more than one group of antihypertensive medications. Additionally, a small proportion of participants with very high TCh were prescribed statins (39%). These subgroups of participants have a very high CVD risk which may not be accurately estimated by QRISK2.[13] This group of patients may need multidisciplinary approach utilising specialist services including genetic testing and cascade screening, which cannot be provided in GP practices.

The second important finding relates to the level of control of risk factors. Despite the substantial improvements in both BP and TCh, values at 18 months were still high. (Figure 2) This indicates that even when patients were started on treatment, there does not seem to be an imperative to ensure that the risk factors are well controlled. This may be due to several factors including high workload of GP practices and the divide between primary and secondary prevention, where for the latter, the targets are much better defined in guidelines. It is also likely that it reflects the way primary care is incentivised i.e. payment is directed for initial

For peer review only - http://bmjopen.bmj.com/site/about/guidelines.xhtml

identification and initiation of treatment rather than for achieving treatment goals. This is further illustrated by the fact that repeated measurements of lipid profile are less frequently performed and less than 50% of subjects with high or very high TCh have a repeated measurements within six months from the NHSHC. This shows lack of adherence to NICE recommendation, which advocates re-examination of lipid profile three months after prescribing statins.[14] Lack of repeated cholesterol measurements may be one of the factors significantly reducing uptake and continuation of statin treatment.

Our findings are consistent with previous research indicating that attendance to NHSHC is associated with increased detection of CVD and risk factors and prescription of therapy.[8, 10, 21] It also confirms the previous findings that the magnitude of reduction in the values of risk factors is larger in high risk groups.[8, 10] Based on the example of high blood pressure management, our study provides strong evidence of the clinical effectiveness of NHSHC as a public programme aimed at reduction of CVD risk. However, the general perception of NHSC should be changed. NHSHC has to be recognised as a sequence of events starting with detection of increased CVD risk during the NHSHC, through prescription of therapy, follow-up monitoring and reassessment needed to decide if further changes to therapy are needed. We have provided new evidence that intervention to improve active participation of patients are needed to achieve expected benefit.

To our knowledge, no specific mechanisms are embedded in the programme to incentivize follow-up, specialist referrals as well as achievement of treatment goals.[22] The NHSHC relies on the mechanism of Quality and Outcome Framework - a voluntary reward and incentive programme rewarding GP practices for the quality of care provided to patients.[23] Our study suggests that this mechanism may be insufficient.

Our data indicate that the problem of patients with moderate and high CVD risk not subjected to follow-up has not been adequately explored. A previous survey study performed in general practices in London in the first year after implementation of the NHSHC identified problems with implementation.[24] The identified problems included non-prescribing of statins to high risk individuals, reluctance to refer to external services and variable patterns of

For peer review only - http://bmjopen.bmj.com/site/about/guidelines.xhtml

organising clinical follow-up with only around 50% of practices organising the recommended annual recall.[24] Forester et al. (2015) also reported that fewer than 52% of patients had repeated monitoring of CVD risk factors over the 15 month period of observation.[8] Several studies reported lower than expected prescriptions of statins to patients diagnosed with elevated TCh and/or high CVD risk during NHSHC.[8-10]

Lower than projected uptake of the NHSHC has been a concern as the major factor influencing cost-effectiveness of the programme.[25] The reason for non-attendance to NHSHC has been extensively investigated using qualitative and quantitative approaches. Recent data indicate steady increase in the uptake now reaching 52%.[16] This threshold of 50% has been commented in the context of sensitivity analysis as satisfactory.[26] Our study indicates a new goal for the research community and Public Health England to work on in the pursuit of improving the overall effectiveness of the NHSHC.

Acknowledgements:

The research team would like to express gratitude to all participant of the GENVASC study. We would also like to thank all healthcare and other professionals involved in performing NHS Health Check as well as research related tasks.

Funding:

The GENVASC study is funded by the NIHR Leicester Biomedical Research Centre (Grant No. BRC-1215-20010)

RD is funded by NIHR – grant number NA

KK is supported by the National Institute for Health Research (NIHR) Applied Research Collaboration East Midlands (ARC EM) and the NIHR Leicester Biomedical Research Centre (BRC) – grant number NA

Conflicts of interest

For peer review only - http://bmjopen.bmj.com/site/about/guidelines.xhtml

KK was a national advisor for the NHS Health Checks Programme

Author contributions

RD - designed the project, performed analysis and is responsible for the overall contents of the manuscript.

VB - designed the project, performed analysis, supervised the statistical analysis and is responsible for the overall contents of the manuscript,

RP- designed the project, performed analysis and is responsible for the overall contents of the manuscript.

NJS - designed the project, performed analysis and is responsible for the overall contents of the manuscript.

CG, RB, SS, SK, MN, AM, AK, AC, AH, KK, NJ, ML, AF were involved in collection and analysis of data

All authors reviewed the content and contributed to the final manuscript.

Data availability:

Data available on request from authors subject to privacy/ethical restrictions.

Research Ethics Approval: Human Participants

The study was approved by the East Midlands - Derby Research Ethics Committee (approval number 12/EM/0208). All study subjects gave informed consent for participation.

Figure Legends:

Figure 1: Weekly incidence of CVDs in relation to NHS Health Check.,

Figure 2: Magnitude of changes in the individual risk factors during follow up.

For peer review only

For peer review only - http://bmjopen.bmj.com/site/about/guidelines.xhtml

References

1. Murray CJ, Richards MA, Newton JN, Fenton KA, Anderson HR, Atkinson C, Bennett D, Bernabe E, Blencowe H, Bourne R, Braithwaite T, Brayne C, Bruce NG, Brugha TS, Burney P, Dherani M, Dolk H, Edmond K, Ezzati M, Flaxman AD, Fleming TD, Freedman G, Gunnell D, Hay RJ, Hutchings SJ, Ohno SL, Lozano R, Lyons RA, Marcenes W, Naghavi M, Newton CR, Pearce N, Pope D, Rushton L, Salomon JA, Shibuya K, Vos T, Wang H, Williams H

C, Woolf AD, Lopez AD, Davis A. UK health performance: findings of the Global Burden of Disease Study 2010. Lancet 2013;**381**(9871):997-1020.
2. Roth GA, Johnson C, Abajobir A, Abd-Allah F, Abera SF, Abyu G, Ahmed M, Aksut B, Alam T, Alam K, Alla F, Alvis-Guzman N, Amrock S, Ansari H, Arnlov J, Asayesh H, Atey TM, Avila-Burgos L, Awasthi A, Banerjee A, Barac A, Barnighausen T, Barregard L, Bedi N, Belay Ketema E, Bennett D, Berhe G, Bhutta Z, Bitew S, Carapetis J, Carrero JJ, Malta DC, Castaneda-Orjuela CA, Castillo-Rivas J, Catala-Lopez F, Choi JY, Christensen H, Cirillo M, Cooper L, Jr., Criqui M, Cundiff D, Damasceno A, Dandona L, Dandona R, Davletov K, Dharmaratne S, Dorairaj P, Dubey M, Ehrenkranz R, El Sayed Zaki M, Faraon EJA, Esteghamati A, Farid T, Farvid M, Feigin V, Ding EL, Fowkes G, Gebrehiwot T, Gillum R, Gold A, Gona P, Gupta R, Habtewold TD, Hafezi-Nejad N, Hailu T, Hailu GB, Hankey G, Hassen HY, Abate KH, Havmoeller R, Hay SI, Horino M, Hotez PJ, Jacobsen K, James S, Javanbakht M, Jeemon P, John D, Jonas J, Kalkonde Y, Karimkhani C, Kasaeian A, Khader Y, Khan A, Khang YH, Khera S, Khoja AT, Khubchandani J, Kim D, Kolte D, Kosen S, Krohn KJ, Kumar GA, Kwan GF, Lal DK, Larsson A, Linn S, Lopez A, Lotufo PA, El Razek HMA, Malekzadeh R, Mazidi M, Meier T, Meles KG, Mensah G, Meretoja A, Mezgebe H, Miller T, Mirrakhimov E, Mohammed S, Moran AE, Musa KI, Narula J, Neal B, Ngalesoni F, Nguyen G, Obermeyer CM, Owolabi M, Patton G, Pedro J, Qato D, Qorbani M, Rahimi K, Rai RK, Rawaf S, Ribeiro A, Safiri S, Salomon JA, Santos I, Santric Milicevic M, Sartorius B, Schutte A, Sepanlou S, Shaikh MA, Shin MJ, Shishehbor M, Shore H, Silva DAS, Sobngwi E, Stranges S, Swaminathan S, Tabares-Seisdedos R, Tadele Atnafu N, Tesfay F, Thakur JS, Thrift A, Topor-Madry R, Truelsen T, Tyrovolas S, Ukwaja KN, Uthman O, Vasankari T, Vlassov V, Vollset SE, Wakayo T, Watkins D, Weintraub R, Werdecker A, Westerman R, Wiysonge CS, Wolfe C, Workicho A, Xu G, Yano Y, Yip P, Yonemoto N, Younis M, Yu C, Vos T, Naghavi M, Murray C. Global, Regional, and National Burden of Cardiovascular Diseases for 10 Causes, 1990 to 2015. J Am Coll Cardiol 2017;**70**(1):1-25.
3. Department of Helath. Putting prevention first: Vascular checks risk assessment and management - impact assessment. 2008 http://data.parliament.uk/DepositedPapers/Files/DEP2008-0910/DEP2008-0910.pdf
4. Department of Health. Economic Modelling for Vascular Checks: Department of Health, 2008
5. Krogsbøll LT, Jørgensen KJ, Gøtzsche PC. Universal health checks should be abandoned. BMJ : British Medical Journal 2013;**347**:f5227.
6. Soljak M, Majeed A, Millett C. Response to Krogsbøll and colleagues: NHS health checks or government by randomised controlled trial? BMJ : British Medical Journal 2013;**347**:f5984.
7. Chang KC, Lee JT, Vamos EP, Soljak M, Johnston D, Khunti K, Majeed A, Millett C. Impact of the National Health Service Health Check on cardiovascular disease risk: a difference-in-differences matching analysis. CMAJ 2016;**188**(10):E228-E238.
8. Forster AS, Dodhia H, Booth H, Dregan A, Fuller F, Miller J, Burgess C, McDermott L, Gulliford MC. Estimating the yield of NHS Health Checks in England: a population-based cohort study. J Public Health (Oxf) 2015;**37**(2):234-40.
9. Caley M, Chohan P, Hooper J, Wright N. The impact of NHS Health Checks on the prevalence of disease in general practices: a controlled study. Br J Gen Pract 2014;**64**(625):e516-21.
10. Kennedy O, Su F, Pears R, Walmsley E, Roderick P. Evaluating the effectiveness of the NHS Health Check programme in South England: a quasi-randomised controlled trial. BMJ Open 2019;**9**(9):e029420.

For peer review only - http://bmjopen.bmj.com/site/about/guidelines.xhtml

11. Cochrane T, Davey R, Iqbal Z, Gidlow C, Kumar J, Chambers R, Mawby Y. NHS health checks through general practice: randomised trial of population cardiovascular risk reduction. BMC Public Health 2012;**12**:944.
12. Public Health England. NHS Health Check Best practice guidance. 2019. https://www.healthcheck.nhs.uk/commissioners-and-providers/national-guidance/
13. Hippisley-Cox J, Coupland C, Vinogradova Y, Robson J, Minhas R, Sheikh A, Brindle P. Predicting cardiovascular risk in England and Wales: prospective derivation and validation of QRISK2. BMJ 2008;**336**(7659):1475-82.
14. The National Institute for Health and Care Excellence (NICE). Cardiovascular disease: risk assessment and reduction, including lipid modification. Clinical guideline [CG181]. 2014. https://www.nice.org.uk/guidance/cg181/chapter/1-recommendations
15. Centere NLBR. https://www2.le.ac.uk/projects/bru/our-research/research-themes/genetics-and-biomarkers/genvasc.
16. Patel R, Barnard S, Thompson K, Lagord C, Clegg E, Worrall R, Evans T, Carter S, Flowers J, Roberts D, Nuttall M, Samani NJ, Robson J, Kearney M, Deanfield J, Waterall J. Evaluation of the uptake and delivery of the NHS Health Check programme in England, using primary care data from 9.5 million people: a cross-sectional study. BMJ Open 2020;**10**(11):e042963.
17. Mancia G, Fagard R, Narkiewicz K, Redon J, Zanchetti A, Bohm M, Christiaens T, Cifkova R, De Backer G, Dominiczak A, Galderisi M, Grobbee DE, Jaarsma T, Kirchhof P, Kjeldsen SE, Laurent S, Manolis AJ, Nilsson PM, Ruilope LM, Schmieder RE, Sirnes PA, Sleight P, Viigimaa M, Waeber B, Zannad F, Redon J, Dominiczak A, Narkiewicz K, Nilsson PM, Burnier M, Viigimaa M, Ambrosioni E, Caufield M, Coca A, Olsen MH, Schmieder RE, Tsioufis C, van de Borne P, Zamorano JL, Achenbach S, Baumgartner H, Bax JJ, Bueno H, Dean V, Deaton C, Erol C, Fagard R, Ferrari R, Hasdai D, Hoes AW, Kirchhof P, Knuuti J, Kolh P, Lancellotti P, Linhart A, Nihoyannopoulos P, Piepoli MF, Ponikowski P, Sirnes PA, Tamargo JL, Tendera M, Torbicki A, Wijns W, Windecker S, Clement DL, Coca A, Gillebert TC, Tendera M, Rosei EA, Ambrosioni E, Anker SD, Bauersachs J, Hitij JB, Caulfield M, De Buyzere M, De Geest S, Derumeaux GA, Erdine S, Farsang C, Funck-Brentano C, Gerc V, Germano G, Gielen S, Haller H, Hoes AW, Jordan J, Kahan T, Komajda M, Lovic D, Mahrholdt H, Olsen MH, Ostergren J, Parati G, Perk J, Polonia J, Popescu BA, Reiner Z, Ryden L, Sirenko Y, Stanton A, Struijker-Boudier H, Tsioufis C, van de Borne P, Vlachopoulos C, Volpe M, Wood DA. 2013 ESH/ESC guidelines for the management of arterial hypertension: the Task Force for the Management of Arterial Hypertension of the European Society of Hypertension (ESH) and of the European Society of Cardiology (ESC). Eur Heart J 2013;**34**(28):2159-219.
18. Piepoli MF, Hoes AW, Agewall S, Albus C, Brotons C, Catapano AL, Cooney MT, Corra U, Cosyns B, Deaton C, Graham I, Hall MS, Hobbs FDR, Lochen ML, Lollgen H, Marques-Vidal P, Perk J, Prescott E, Redon J, Richter DJ, Sattar N, Smulders Y, Tiberi M, van der Worp HB, van Dis I, Verschuren WMM, Binno S, Group ESCSD. 2016 European Guidelines on cardiovascular disease prevention in clinical practice: The Sixth Joint Task Force of the European Society of Cardiology and Other Societies on Cardiovascular Disease Prevention in Clinical Practice (constituted by representatives of 10 societies and by invited experts)Developed with the special contribution of the European Association for Cardiovascular Prevention & Rehabilitation (EACPR). Eur Heart J 2016;**37**(29):2315-2381.
19. The National Institute for Health and Care Excellence (NICE). Chronic kidney disease in adults: assessment and management. Clinical guideline [CG182]. 2014. https://www.nice.org.uk/guidance/cg182
20. StataCorp. Stata Statistical Software: Release 16. College Station, TX: StataCorp LLC. 2019.
21. Forster AS, Burgess C, Dodhia H, Fuller F, Miller J, McDermott L, Gulliford MC. Do health checks improve risk factor detection in primary care? Matched cohort study using electronic health records. J Public Health (Oxf) 2016;**38**(3):552-559.
22. Department of Health . Putting Prevention First, Vascular Check: Risk Assessment and Management. London: Department of Health; 2008. http://data.parliament.uk/DepositedPapers/Files/DEP2008-0910/DEP2008-0910.pdf

For peer review only - http://bmjopen.bmj.com/site/about/guidelines.xhtml

23. Digital NHS. Quality and Outcomes Framework (QOF), enhanced services and core contract extraction specifications (business rules). https://digital.nhs.uk/data-and-information/data-collections-and-data-sets/data-collections/quality-and-outcomes-framework-qof.
24. Nicholas JM, Burgess C, Dodhia H, Miller J, Fuller F, Cajeat E, Gulliford MC. Variations in the organization and delivery of the 'NHS health check' in primary care. J Public Health (Oxf) 2013;**35**(1):85-91.
25. Martin A, Saunders CL, Harte E, Griffin SJ, MacLure C, Mant J, Meads C, Walter FM, Usher-Smith JA. Delivery and impact of the NHS Health Check in the first 8 years: a systematic review. Br J Gen Pract 2018;**68**(672):e449-e459.
26. Newton JN, Thompson K. NHS Health Check: national evaluation findings and implications. CMAJ 2017;**189**(4):E172.

For peer review only

For peer review only - http://bmjopen.bmj.com/site/about/guidelines.xhtml

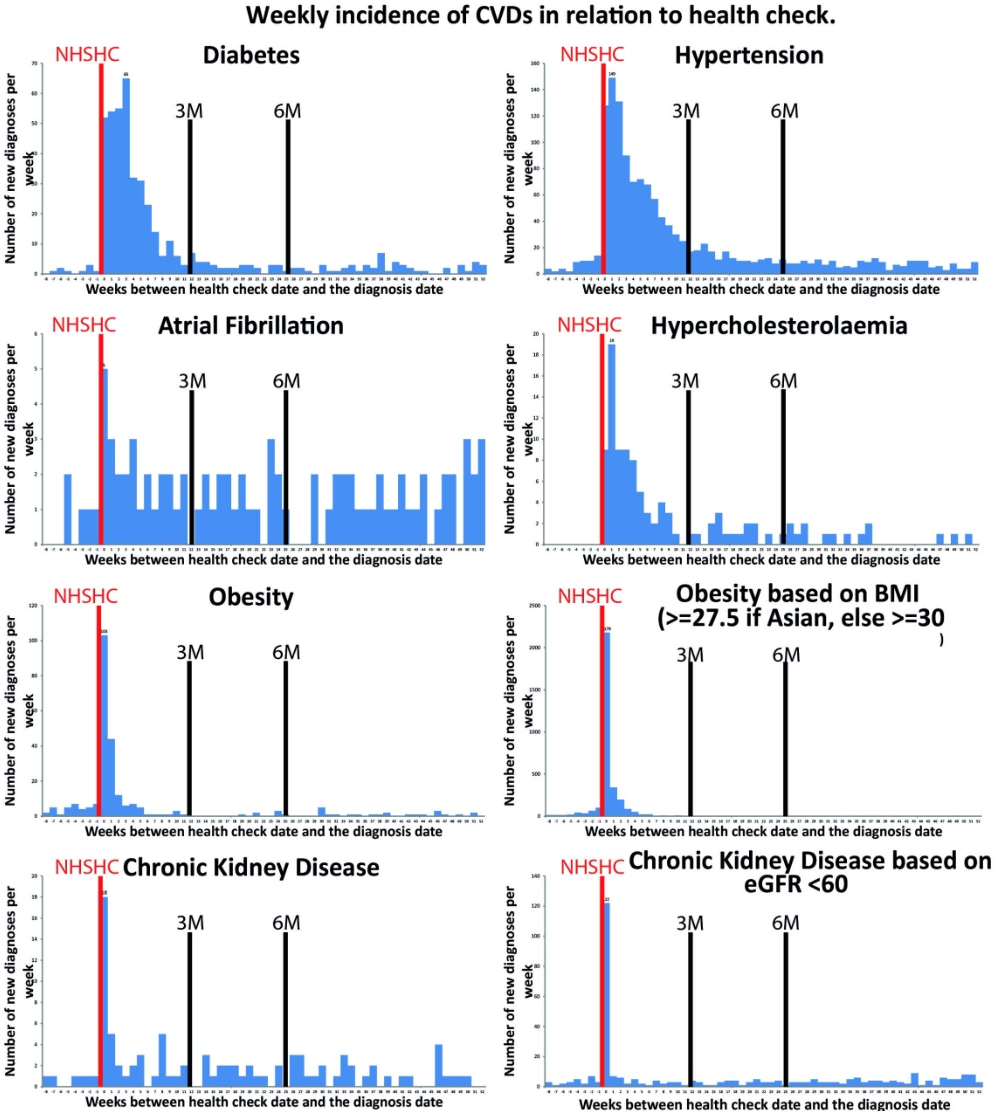

Weekly incidence of CVD in relation to health check

198x222mm (300 x 300 DPI)

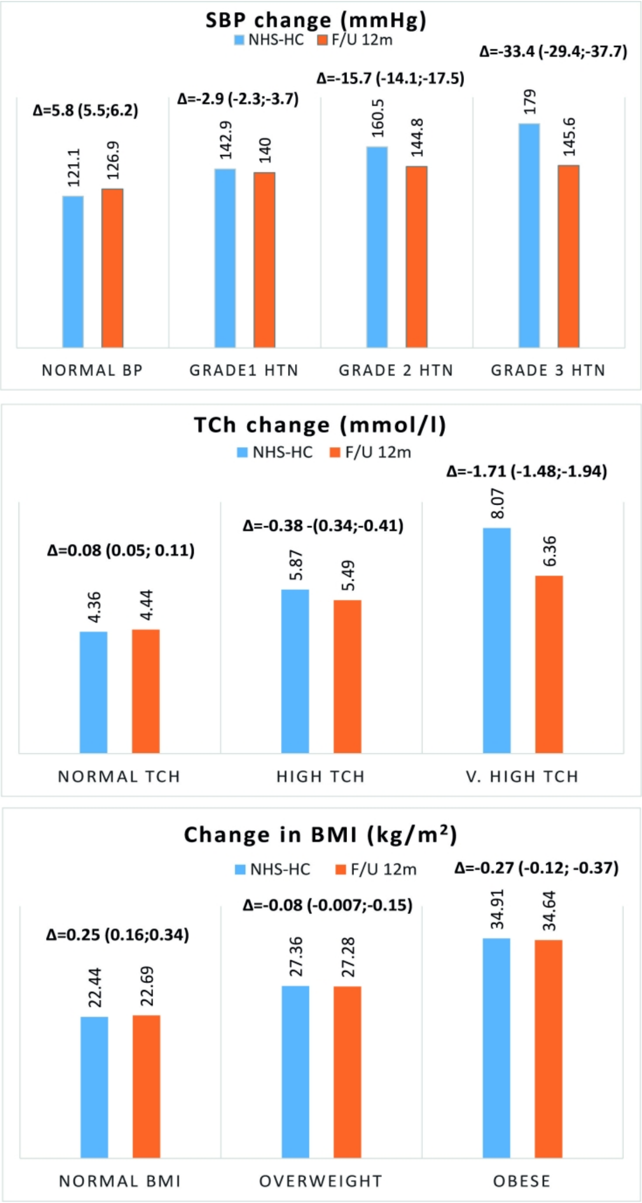

Magnitude of change in the individual risk factors

156x291mm (300 x 300 DPI)

SUPPLEMENTARY DATA

Clinical variables and measurements

BP was measured individually in GP practices using standardised oscillatory BP measuring devices after a minimum of 5-minute rest. Total Cholesterol (TC, mmol/l), High Density Lipoprotein-cholesterol (HDL-C, mmol/l) and serum creatinine (umol/l) were measured in accredited local laboratories following standard procedures. Non-HDL cholesterol (non-HDL-C, mmol/l) was calculated by subtracting value of HDL cholesterol from TC, Low Density Lipoprotein-cholesterol (LD-CL, mmol/l) was calculated using the Friedewald equation and estimated glomerular filtration rate (eGFR, ml/min/1.73xm^2) was calculated using the MDRD formula.

Townsend Deprivation Score

The Townsend Score is a composite measure of material deprivation of the population in UK. The Index was first introduced in 1987. The Townsend Score is published by the Office of National Statistics. Townsend score is calculated using four census variables for any given geographical area. These variables include: unemployment - defined as the percentage of economically active residents who are unemployed; non-car ownership measuring the number of households without a car; non-home ownership measuring the number of households renting and owning their accommodations; overcrowding – measuring whether the accommodation is suitable in size for the number of occupants. The Townsend Deprivation Scores were calculated using percentages of the weighted and normalised (where appropriate) four previously listed indicators. Townsend Deprivation Scores are usually split into quintiles to provide map visualisations of deprivation and to assess the spread of deprivation across areas.

https://statistics.ukdataservice.ac.uk/dataset/2011-uk-townsend-deprivation-scores

For peer review only - http://bmjopen.bmj.com/site/about/guidelines.xhtml

Supplementary Table 1. Participants' baseline characteristics according to CVD risk levels at the initial Health Check.

		CVD risk			
	All participants n=27,888	Low QRISK2 <10% n=21,636 (78)	High QRISK2 10%-20% n=5,090 (18)	Very high QRISK2>20% n=1,162 (4)	Data Coverage (%)
**Demographic**					
Males, n (%)	12,322 (44)	8,206 (38)	3,118 (61)	998 (86)	100.0
Age, years	51 (44; 60)	48 (43; 54)	65 (60; 69)	69 (64; 72)	100.0
Ethnicity, n (%)					99.5
White	22,677 (81)	17,212 (80)	4,477 (88)	988 (85)	
Asian	3,686 (13)	3,030 (14)	499 (9.8)	157 (13)	
Black	535 (1.9)	501 (2.3)	33 (0.7)	1 (0.1)	
Other ethnicity	990 (3.6)	893 (4.1)	81 (1.6)	16 (1.4)	
Quintiles of deprivation, n (%)					57.5
1st quintile (least deprived)	4,757 (30)	3,779 (30)	821 (28)	157 (24)	
2nd quintile	3,302 (21)	2,585 (21)	605 (21)	112 (17)	
3rd quintile	2,523 (15)	1,939 (16)	488 (16)	96 (14)	
4th quintile	2,070 (13)	1,598 (13)	378 (13)	94 (14)	
5th quintile (most deprived)	3,389 (21)	2,533 (20)	646 (22)	210 (31)	
**Clinical**					
Smoking					82.6
Non-smoker	13,810 (60)	11,416 (64)	2,081 (49)	313 (31)	
Ex-smoker	5,415 (23)	3,738 (21)	1,304 (31)	373 (38)	
Current smoker	3,813 (17)	2,673 (15)	834 (20)	306 (31)	
Obesity					
BMI, kg/m^2	27.9 (5.1)	27.1 (5.1)	27.5 (5.0)	28.2 (5.1)	100.0
Abnormal body weight (BMI≥25, BMI>23for Asian subjects), n (%)	18,443 (66.1)	14,099 (65.2)	3,458 (67.9)	886 (76.2)	
Blood pressure					

For peer review only - http://bmjopen.bmj.com/site/about/guidelines.xhtml

SBP, mmHg	127.6 (16.0)	124.9 (14.7)	135.6 (15.9)	143.6 (18.9)	100.0
DBP, mmHg	78.8 (10.0)	78.1 (9.7)	80.6 (10.4)	83.4 (11.7)	97.0
Elevated BP (SBP≥140mmHg and/or DBP≥90mmHg), n (%)	7,005 (25.1)	4,251 (19.7)	2,079 (40.8)	675 (58.1)	
Cholesterol					
TCh, mmol/l	5.29 (0.97)	5.22 (0.95)	5.53 (0.99)	5.53 (1.04)	81.5
Non-HDL, mmol/l	3.76 (0.96)	3.67 (0.94)	3.98 (0.96)	4.17 (1.00)	44.0
Elevated TCh (TCh≥5mmol/l), n (%)	14,140 (62.2)	10,449 (59.3)	3,016 (71.9)	675 (72.4)	
eGFR					
eGFR, ml/min/1.73m^2	90 (83, 90)	90 (85; 90)	90 (79; 90)	87 (78; 90)	73.5
Abnormal eGFR (eGFR<60 ml/min/1.73m^2), n (%)	174 (0.9)	94 (0.6)	60 (1.6)	20 (2.3)	

Results are mean (SD) or median (1st quartile; 3rd quartile) unless otherwise stated; Data coverage refers to the proportion of subjects with available data. **Abbreviations:** BMI-body mass index; SBP-systolic blood pressure; DBP-diastolic blood pressure; TCh-total cholesterol; Quintiles of deprivation were calculated using the Townsend Index of Deprivation 2019.[27] For the calculation of Qrisk2, Townsend index of deprivation was substituted with the average value of the index (i.e. "0") in participants with missing data.

For peer review only - http://bmjopen.bmj.com/site/about/guidelines.xhtml

Supplementary Figure 1. Geographical distribution of recruitment to the GENVASC study

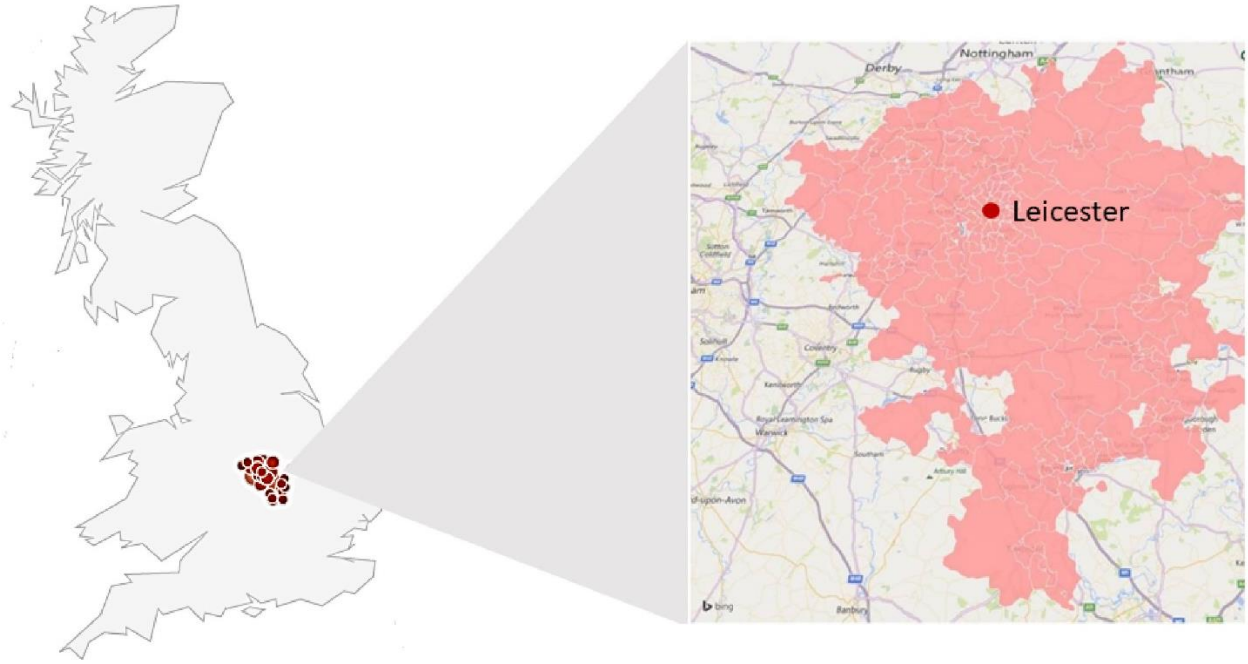

For peer review only

For peer review only - http://bmjopen.bmj.com/site/about/guidelines.xhtml

STROBE 2007 (v4) Statement—Checklist of items that should be included in reports of *cohort studies*

Section/Topic	Item #	Recommendation	Reported on page #
**Title and abstract**	1	(*a*) Indicate the study's design with a commonly used term in the title or the abstract	1,3
		(*b*) Provide in the abstract an informative and balanced summary of what was done and what was found	3
**Introduction**			
Background/rationale	2	Explain the scientific background and rationale for the investigation being reported	5
Objectives	3	State specific objectives, including any prespecified hypotheses	5
**Methods**			
Study design	4	Present key elements of study design early in the paper	5-9
Setting	5	Describe the setting, locations, and relevant dates, including periods of recruitment, exposure, follow-up, and data collection	5-9
Participants	6	(*a*) Give the eligibility criteria, and the sources and methods of selection of participants. Describe methods of follow-up	6
		(*b*) For matched studies, give matching criteria and number of exposed and unexposed	NA
Variables	7	Clearly define all outcomes, exposures, predictors, potential confounders, and effect modifiers. Give diagnostic criteria, if applicable	7-8
Data sources/ measurement	8*	For each variable of interest, give sources of data and details of methods of assessment (measurement). Describe comparability of assessment methods if there is more than one group	5-9
Bias	9	Describe any efforts to address potential sources of bias	7
Study size	10	Explain how the study size was arrived at	6
Quantitative variables	11	Explain how quantitative variables were handled in the analyses. If applicable, describe which groupings were chosen and why	8-9
Statistical methods	12	(*a*) Describe all statistical methods, including those used to control for confounding	8-9
		(*b*) Describe any methods used to examine subgroups and interactions	8-9
		(*c*) Explain how missing data were addressed	8-9
		(*d*) If applicable, explain how loss to follow-up was addressed	8-9
		(*e*) Describe any sensitivity analyses	8-9
**Results**			

For peer review only

Participants	13*	(a) Report numbers of individuals at each stage of study—eg numbers potentially eligible, examined for eligibility, confirmed eligible, included in the study, completing follow-up, and analysed	9-14
		(b) Give reasons for non-participation at each stage	9-14
		(c) Consider use of a flow diagram	
Descriptive data	14*	(a) Give characteristics of study participants (eg demographic, clinical, social) and information on exposures and potential confounders	9-10, supplementary materials
		(b) Indicate number of participants with missing data for each variable of interest	Table 1, Table 2, Table 3
		(c) Summarise follow-up time (eg, average and total amount)	11-12
Outcome data	15*	Report numbers of outcome events or summary measures over time	9-14
Main results	16	(*a*) Give unadjusted estimates and, if applicable, confounder-adjusted estimates and their precision (eg, 95% confidence interval). Make clear which confounders were adjusted for and why they were included	9-14, Figures
		(*b*) Report category boundaries when continuous variables were categorized	9-14, Tables 1-3
		(*c*) If relevant, consider translating estimates of relative risk into absolute risk for a meaningful time period	NA
Other analyses	17	Report other analyses done—eg analyses of subgroups and interactions, and sensitivity analyses	8
**Discussion**			
Key results	18	Summarise key results with reference to study objectives	14-16
**Limitations**			
Interpretation	20	Give a cautious overall interpretation of results considering objectives, limitations, multiplicity of analyses, results from similar studies, and other relevant evidence	14-16
Generalisability	21	Discuss the generalisability (external validity) of the study results	4- strengths and limitations
**Other information**			
Funding	22	Give the source of funding and the role of the funders for the present study and, if applicable, for the original study on which the present article is based	17

*Give information separately for cases and controls in case-control studies and, if applicable, for exposed and unexposed groups in cohort and cross-sectional studies.

Note: An Explanation and Elaboration article discusses each checklist item and gives methodological background and published examples of transparent reporting. The STROBE checklist is best used in conjunction with this article (freely available on the Web sites of PLoS Medicine at http://www.plosmedicine.org/, Annals of Internal Medicine at http://www.annals.org/, and Epidemiology at http://www.epidem.com/). Information on the STROBE Initiative is available at www.strobe-statement.org.

For peer review only

BMJ Open

Evaluating the clinical effectiveness of the NHS Health Check programme: A prospective analysis in the Genetics and Vascular Health Check (GENVASC) Study

Journal:	*BMJ Open*
Manuscript ID	bmjopen-2022-068025.R1
Article Type:	Original research
Date Submitted by the Author:	28-Mar-2023

Complete List of Authors:	Debiec, Radoslaw; University of Leicester, Department of Cardiovascular Sciences and NIHR Cardiovascular Research Centre<br>Lawday, Daniel; University of Leicester, Department of Cardiovascular Sciences and NIHR Cardiovascular Research Centre<br>Bountziouka, Vasiliki; University of Leicester, Department of Cardiovascular Sciences and NIHR Cardiovascular Research Centre; University of the Aegean, Department of Computer simulations, Genomics and Data analysis lab, Department of Food Science and Nutrition, University of the Aegean,<br>Beeston, Emma; University of Leicester, Department of Cardiovascular Sciences and NIHR Cardiovascular Research Centre<br>Greengrass, Chris; University of Leicester, Department of Cardiovascular Sciences and NIHR Cardiovascular Research Centre<br>Bramley, Richard; University of Leicester, Department of Cardiovascular Sciences and NIHR Cardiovascular Research Centre<br>Sehmi, Sue; University of Leicester, Department of Cardiovascular Sciences and NIHR Cardiovascular Research Centre<br>Kharodia, Shireen; University of Leicester, Department of Cardiovascular Sciences and NIHR Cardiovascular Research Centre<br>Newton, Michelle; University of Leicester, Department of Cardiovascular Sciences and NIHR Cardiovascular Research Centre<br>Marshall, Andrea; University of Leicester, Department of Cardiovascular Sciences and NIHR Cardiovascular Research Centre<br>Krzeminski, Andre; Albany House Medical Centre<br>Zafar, Azhar; University of Leicester, Diabetes Research Centre; Diabetes and Cardiovascular Medicine General Practice Alliance Federation Research and Training Academy<br>Chahal, Anuj; South Leicestershire Medical Group<br>Heer, Amardeep; Lakeside Healthcare Research<br>Khunti, Kamlesh; University of Leicester, Diabetes Research Centre<br>Joshi, Nitin; Willowbrook Medical Centre<br>Lakhani, Mayur; University of Leicester, Department of Health Sciences<br>Farooqi, Azhar; NIHR Applied Research Collaboration<br>Patel, Riyaz; University College London, Institute of Cardiovascular Science<br>Samani, Nilesh; University of Leicester, Department of Cardiovascular Sciences and NIHR Cardiovascular Research Centre

**Primary Subject Heading**:	Cardiovascular medicine
Secondary Subject Heading:	Epidemiology, Evidence based practice, General practice / Family practice, Health policy
Keywords:	PREVENTIVE MEDICINE, Health policy < HEALTH SERVICES ADMINISTRATION & MANAGEMENT, Primary Prevention, Cardiology < INTERNAL MEDICINE

I, the Submitting Author has the right to grant and does grant on behalf of all authors of the Work (as defined in the below author licence), an exclusive licence and/or a non-exclusive licence for contributions from authors who are: i) UK Crown employees; ii) where BMJ has agreed a CC-BY licence shall apply, and/or iii) in accordance with the terms applicable for US Federal Government officers or employees acting as part of their official duties; on a worldwide, perpetual, irrevocable, royalty-free basis to BMJ Publishing Group Ltd ("BMJ") its licensees and where the relevant Journal is co-owned by BMJ to the co-owners of the Journal, to publish the Work in this journal and any other BMJ products and to exploit all rights, as set out in our licence.

The Submitting Author accepts and understands that any supply made under these terms is made by BMJ to the Submitting Author unless you are acting as an employee on behalf of your employer or a postgraduate student of an affiliated institution which is paying any applicable article publishing charge ("APC") for Open Access articles. Where the Submitting Author wishes to make the Work available on an Open Access basis (and intends to pay the relevant APC), the terms of reuse of such Open Access shall be governed by a Creative Commons licence – details of these licences and which Creative Commons licence will apply to this Work are set out in our licence referred to above.

Other than as permitted in any relevant BMJ Author's Self Archiving Policies, I confirm this Work has not been accepted for publication elsewhere, is not being considered for publication elsewhere and does not duplicate material already published. I confirm all authors consent to publication of this Work and authorise the granting of this licence.

For peer review only

Evaluating the clinical effectiveness of the NHS Health Check programme: A prospective analysis in the Genetics and Vascular Health Check (GENVASC) Study

Running title: Clinical effectiveness of the NHS Health Check programme

Radoslaw Debiec[a], Daniel Lawday[a], Vasiliki Bountziouka[a,b], Emma Beeston[a], Chris Greengrass[a], Richard Bramley[a], Sue Sehmi[a], Shireen Kharodia[a], Michelle Newton[a], Andrea Marshall[a], Andre Krzeminski[c], Azhar Zafar[d,e], Anuj Chahal[f], Amardeep Heer[g], Kamlesh Khunti[d], Nitin Joshi[h], Mayur Lakhani[i], Azhar Farooqi[i], Riyaz Patel[j], Nilesh J Samani[a]

Affiliations of authors:

a) Department of Cardiovascular Sciences, University of Leicester and NIHR Cardiovascular Research Centre, Glenfield Hospital, Leicester, UK

b) Computer simulations, Genomics and Data analysis lab, Department of Food Science and Nutrition, University of the Aegean, Lemnos, Greece

c) Albany House Medical Centre, Wellingborough, UK

d) Diabetes Research Centre, University of Leicester, UK

e) Diabetes and Cardiovascular Medicine General Practice Alliance Federation Research and Training Academy, UK

f) South Leicestershire Medical Group, Kibworth Beauchamp, UK

g) Lakeside Healthcare, Corby, UK

h) Willowbrook Medical Centre, Leicester, UK

i) Department of Health Sciences, University of Leicester, UK

j) Institute of Cardiovascular Science, University College London, London, UK

Address for correspondence:

Dr Radoslaw Debiec,

Department of Cardiovascular Sciences and NIHR Leicester Biomedical Research Centre,

Glenfield Hospital,

For peer review only

For peer review only - http://bmjopen.bmj.com/site/about/guidelines.xhtml

Groby Road, LE39QP

rmd24@leicester.ac.uk

Tel: 01162044789

Disclaimers: KK was a national advisor for the NHS Health Checks Programme

Word Count: 4096

Sources of support

The GENVASC study is funded by the NIHR Leicester Biomedical Research Centre (Grant No. BRC-1215-20010)

For peer review only

For peer review only - http://bmjopen.bmj.com/site/about/guidelines.xhtml

Abstract

Objective:

The aim of the study was to assess the clinical effectiveness of the national Cardiovascular Disease (CVD) prevention program - NHS Health Check (NHSHC) in reduction of CVD risk.

Design:

Prospective cohort study.

Setting:

147 General Practices (GP) in Leicestershire and Northamptonshire in England, UK.

Participants:

27,888 individuals undergoing NHSHC with a minimum of 18 months of follow up data.

The outcome measures:

The primary outcomes were NHSHC attributed detection of CVD risk factors, prescription of medications, changes in values of individual risk factors and frequency of follow up.

Results:

At recruitment, 18% of participants had high CVD risk (10-20% 10-year risk) and 4% very high CVD risk (>20% 10-year risk). New diagnoses or hypertension was made in 2.3% participants, hypercholesterolaemia in 0.25%, and diabetes mellitus in 0.9%. New prescription of stains and anti-hypertensive medications was observed in 5.4% and 5.4% of participants, respectively. Total cholesterol was decreased on average by 0.38 mmol/l (95% CI: -0.34; -0.41) and 1.71 mmol/l (-1.48; -1.94) in patients with initial cholesterol >5mmol/l and >7.5mmol/l, respectively. Systolic blood pressure was decreased on average by 2.9 mmHg (-2.3; -3.7), 15.7 mmHg (-14.1; -17.5) and 33.4 mmHg (-29.4; -37.7), in patients with grade one, two and three hypertension, respectively. About 1 out of 3 patients with increased CVD risk had no record of follow-up or treatment.

Conclusions:

Majority of patients identified with increased CVD risk through the NHSHC were followed-up and received effective clinical interventions. However, one third of high CVD risk patients had

For peer review only

For peer review only - http://bmjopen.bmj.com/site/about/guidelines.xhtml

no follow-up and therefore did not receive any treatment. Our study highlights areas of focus which could improve the effectiveness of the programme.

Trial registration:

GENVASC ClinicalTrials.gov Identifier: NCT04417387

Key words:

NHS health check, primary prevention, cardiovascular, clinical effectiveness

Strengths and limitations of the study

- Prospective observation of large cohort of patients from 147 GP Practices undergoing NHSHC
- Purposefully built, comprehensive database
- Possible selection bias – individuals volunteering to take part in the study might represent more "proactive" subgroup of NHSHC attendees
- Larger than national average proportion of non-white ethnicities representing population structure of the region
- The performed analysis focused on CVD risk factors recorded in the GPs record but has not covered influence of NHSHC on lifestyle: physical exercise, diet or alcohol consumption

For peer review only - http://bmjopen.bmj.com/site/about/guidelines.xhtml

Introduction

Cardiovascular disease (CVD) is a leading cause of premature morbidity and mortality in England.[1, 2] To address this, in 2009 the UK government introduced first in the world rolling national CVD prevention programme, delivered by the National Health Service (NHS). Now known as the NHS Health Check (NHSHC), the programme aims to identify and treat the main risk factors driving CVD, among people aged 40-74 years, who were otherwise well.[3] It was estimated through economic modelling that NHSHC could prevent over 1600 myocardial infarctions and strokes and 650 premature CVD deaths annually.[4]

The absence of randomised clinical trials demonstrating the effectiveness of such a programme, has continued to raise debate about its value.[5, 6] Instead, several studies sought to assess the effectiveness of the NHSHC using various observational datasets, but often with conflicting results, casting further doubt about the ability of the programme to achieve the stated goals.[7-11]

The Genetics and Vascular Health Check study (GENVASC ClinicalTrials.gov Identifier: NCT04417387) was instigated in 2012 to assess the value of adding a polygenic risk score (PRS) for CVD to the NHSHC for prediction of cardiovascular events. The study recruited patients attending for the NHSHC in 147 General Practices in two counties in England (Leicestershire and Northamptonshire) and collected comprehensive clinical data comprising the NHSHC as well as subsequent follow-up visits. This dataset now permits evaluation of the impact of NHSHC in more detail than previously reported. In particular, the dataset allows for detailed analysis of the sequelae of detecting a risk factor, prescription of pharmacotherapy, clinical follow-up and their correlation with observed reduction in the risk factor. Specifically, we were able to fully characterise the subpopulation of individuals, who attended the initial NHSHC and were identified to have high and very high cardiovascular risk as well as individuals with gross elevations of individual risk factors. The main aim of our study

For peer review only - http://bmjopen.bmj.com/site/about/guidelines.xhtml

was to assess effectiveness of clinical interventions and identify factors that may influence clinical and cost effectiveness of the NHSHC.

Methods

NHSHC

The NHSHC is performed during a dedicated primary care visit and involves structured assessment of CVD risk.[12] Adults aged 40-74 years, without pre-existing CVD, diabetes mellitus, hypertension (HTN), hypercholesterolaemia and not taking statins are invited to attend the programme. The 10-year risk of developing CVD is calculated using QRISK2 score.[13] The attendees receive consultation regarding their CVD risk and individual risk factors: body mass index (BMI), tobacco smoking, blood pressure (BP), cholesterol level, alcohol intake, physical activity and risk of diabetes. Patients found to have increased overall CVD risk (QRISK2 score≥10%) or elevated individual risk factor values are given lifestyle modification advice, pharmacological treatment[14] and/or are referred to specialist services. Subjects with QRISK2<10% are invited for re-assessment every 5 years.

GENVASC study

GENVASC is a large observational study run in conjunction with Clinical Commissioning Groups and Primary Care practices in UK and coordinated by the NIHR Leicester Biomedical Research Centre. The GENVASC study recruited multi ethnic individuals aged 40-74 years, attending the NHSHC at any of the 147 participating General Practitioners' (GP) practices in Leicestershire and Northamptonshire, UK. **(Supplementary Figure 1)** The main aim of the study is to evaluate the additional clinical value of a PRS on top of traditional risk scores in prediction of subsequent CVD events.[15] Participants of the GENVASC are followed up using primary care databases. The collected information includes clinical diagnoses, laboratory and imaging tests, hospital admissions, and referrals to external services as well as medicinal prescriptions.

Study Participants

For peer review only - http://bmjopen.bmj.com/site/about/guidelines.xhtml

For the purpose of current analysis we included 27,888 participants of the GENVASC study recruited between 17/06/2010 and 04/09/2018 who had 18 months of follow-up data.[15] Based on an estimate from Patel et al.[16] about the annual attendance to NHSHSC in Leicestershire and Northamptonshire, the population used for current analysis constituted around 10% of all NHSHC attendees in these two counties. All participants gave their consent to access their medical records during the duration of the study.

<u>Clinical Variables and definitions</u>

For details regarding methods of assessment and extraction of clinical and laboratory variables see Supplementary Material. Overall, 10-year CVD risk was defined from QRISK2 score and categorised as low (<10%), high (10-20%) and very high (>20%).[13] Blood pressure (BP) was defined as normal if systolic blood pressure (SBP) <140mmHg and/or diastolic blood pressure (DBP) <90mmHg; grade one hypertension (HTN) if SBP was 140-159mmHg and/or DBP 90-99mmHg, grade two HTN if SBP was 160-179mmHg and/or DBP 100-109mmHg, grade three HTN if SBP was≥180mmHg and/or DBP≥110mmHg.[17] Total cholesterol (TCh) was defined as normal for values <5.0mmol/l, high for TCh values 5.0-7.49mmol/l and very high for TCh ≥7.5mmol/l.[18] Non-high density lipoprotein (non-HDL) was defined as normal if values were <3.8mmol/l and high for values ≥3.8mmol/l.[18] Kidney function was considered normal if eGFR≥60ml/min/1.73xm^2; chronic kidney disease (CKD) was diagnosed if eGFR<60ml/min/1.73xm^2.[19] Body weight was assessed using body mass index (BMI) and considered as healthy body weight if BMI <25kg/m^2 (<23 kg/m^2 for South Asians), overweight for BMI from 25 to 29.9kg/m^2 (23 to 27.4 kg/m^2 for South Asians) and obese if BMI ≥30 kg/m^2 (>27.5 for South Asians).

Baseline clinical observations were defined as measurements taken during the NHSHC or the nearest measurement taken around the date of NHSHC but within 30 days from the date of the check. Any new diagnoses of CVD risk factors made from the date of NHSHC to 12 weeks after were attributed to the NHSHC visit. This 12-week period, although

For peer review only

For peer review only - http://bmjopen.bmj.com/site/about/guidelines.xhtml

arbitrary, was considered appropriate for making clinical diagnosis and starting any treatment and supported by the bar plot of distribution of weekly diagnoses post NHSHC. **(Figure 1)**

Outcomes of interest

The assessment of the impact of the NHSHC was performed using the following measures:

- risk factor detection – absolute number and proportion of participants with an abnormal values of BP, TCh, eGFR, BMI, current tobacco smoking or having a clinical diagnosis of HTN, diabetes, hypercholesterolaemia, CKD, or atrial fibrillation. Both the rates of clinically coded diagnoses and the rates of observed abnormal values were presented to accurately assess prevalence of risk factors.
- medication prescription and non-medical interventions (e.g. smoking cessation) – absolute number and proportion of subjects started on a given therapy
- frequency of follow-up – number of clinical visits/reviews within the specified periods
- change in values of individual risk factors – difference between the value of a given clinical characteristic recorded during the NHSHC and first value of this characteristic recorded after 12 months from the NHSHC visit

Data Extraction

Data about participants were extracted from the GP information technology systems. The Primary care databases (EMIS and SystmOne) were used to obtain participants' socio-demographic information (age, sex, and ethnicity), anthropometric measurements (height, weight), information about health-related behaviour (tobacco smoking), clinical diagnoses, blood results and prescribed medicines. Relevant data were extracted using NHS Numbers, SNOMED CT/ DM&D and Clinical Terms Version 3 (CTV3) (formerly known as the Read codes).

For peer review only - http://bmjopen.bmj.com/site/about/guidelines.xhtml

Statistical analysis

Categorical variables (sex, ethnicity, deprivation based on quintiles of Townsend 2011 index (for further information on Townsend index see Supplementary Data – Townsend Deprivation Score), smoking status, BP category, TCh category, renal function category, overweight/ obesity, QRISK2 category) are shown as frequencies (relative frequency) whilst continuous data are shown as means and standard deviation (SD) if normally distributed (SBP, DBP, TCh), or median (interquartile range (IQR)) otherwise (age, eGFR). Normality was assessed using graphical methods (i.e. histograms, P-P and Q-Q plots).

Group comparisons were performed using a chi-square test for categorical variables and the one-way ANOVA for the normally distributed continuous variables or the Kruskall-Wallis for the non-normally distributed ones. The change in the levels of individual risk factors was calculated as the difference between the initial NHSHC measurement and the measurement during follow-up, after a period of twelve months. To assess the statistical significance of that comparison, the paired t-test for the continuous and the McNemar test for the nominal data were used.

Subjects with no repeated values recorded for the measured variables from after the initial NHSHC visit to the total time of follow-up observation of 18 months were considered as not-followed up. The comparisons between individuals without follow up and those that were followed up were performed with the independent t-test and Pearson's chi-square test for the continuous and nominal data respectively. A logistic regression model was used to assess whether socio-demographic (age, sex, deprivation, ethnicity) and clinical factors (SBP, DBP, TCh, BMI, smoking) were associated with the absence of follow-up. Significance level was set to 0.05, all tests were two-sided and Stata v16.0 was used for all the calculations.[20]

Patient and public involvement

GENVASC was developed in collaboration with the NIHR Leicester Biomedical Research Centre Cardiovascular Public Involvement Group. The research plan and all public facing materials were reviewed and approved prior to ethics submission. The group were

For peer review only - http://bmjopen.bmj.com/site/about/guidelines.xhtml

updated biannually on the study. This was later extended to our tissue public involvement group (The Exceed Group).

Results

Key characteristics of the study cohort

Overall 27,888 GENVASC participants were included in the current analysis. The median age of included participants was 51 years, 44% (n=12,322) were men, 81% (n=22,677) were of white ethnicity and 13% (n=3,686) were South Asians. (**Supplementary Table 1**) The GENVASC population was, in general younger, had a higher proportion of Asians amongst the ethnic minorities, and had more participants from the extremes of the Townsend deprivation index (quintiles 1 and 5), compared to the NHSHC attendees across England or the general adult population aged between 40-74 years, albeit the differences were small. (**Table 1**)

Table 1. Comparison of GENVASC study participants included in the current analysis to the general population of England and large population of NHSHC attendees.

	ONS data*<br>n-22,805,612	NHSHC data*<br>n=5,102,758	GENVASC study<br>n=27,888	Missingness<br>(%)
**Demographic**				
Males, n (%)	11,200,690 (49.1)	2 311 604 (45.3)	12,327 (44.3)	-
Age bands (years)				-
40-49	7,525,814 (33)	1,951,264 (38.2)	12,256 (43.9)	
50-59	7,089,322(31.1)	1,742,003 (34.1)	8,495 (30.5)	
60-74	8,190,476 (35.9)	1,409,491 (27.7)	7,137 (25.6)	
Ethnicity, n (%)				-
White	20,383,677 (89.3)	4,067,864 (79.7)	22,677 (81.3)	
Asian	1,341,580 (5.9)	368,145 (7.2)	3,686 (13.2)	
Black	585,756 (2.6)	148,160 (2.9)	535 (1.9)	

For peer review only - http://bmjopen.bmj.com/site/about/guidelines.xhtml

Other ethnicity	494,599 (2.2)	142,621 (2.8)	990 (3.6)	
Missing data		375,968 (7.4)		
Quintiles of deprivation, n (%)				2.4
1st quintile (least deprived)	4,996,212 (21.9)	1,129,670 (22.1)	8,332 (29.9)	
2nd quintile	4,901,834 (21.5)	1,094,925 (21.5)	5,867 (21.0)	
3rd quintile	4,707,382 (20.6)	1,027,096 (20.1)	4,429 (15.9)	
4th quintile	4,286,645 (18.8)	954,656 (18.7)	3,415 (12.3)	
5th quintile (most deprived)	3,913,539 (17.2)	893,194 (17.5)	5,167 (18.5)	
Missing data	-	3217 (0.1)	678 (2.4)	

ONS - Office for National Statistics, NHSHC – NHS Health Check, GENVASC - The Genetics and Vascular Health Check study,

* Patel R, Barnard S, Thompson K, Lagord C, et al. Evaluation of the uptake and delivery of the NHS Health Check programme in England, using primary care data from 9.5 million people: a cross-sectional study. BMJ Open 2020;**10**(11):e042963.

** The presented Townsend scores were obtained using post codes provided by patients on consent forms (available for only a proportion of patients). All presented QRISK2 scores incorporate Townsend scores (available to GPs at the time of consultation)

Cardiovascular risk groups

At recruitment, 18% (n=5,090) of participants were classified to have high CVD risk and 4% (n=1,162) to have very high CVD risk. **(Supplementary Table 1).** Patients in the high and very high CVD risk groups were on average older than patients in the low CVD risk groups (median age 65, 69 and 48 years, respectively), were more like to be male, of white ethnic origin and current smokers. **(Supplementary Table 1)**

Diagnoses and risk factor detection

The majority of new clinical diagnoses of HTN, hypercholesterolaemia, diabetes mellitus and CKD occurred within the first 12 weeks following attendance to the NHSHC.

For peer review only - http://bmjopen.bmj.com/site/about/guidelines.xhtml

For peer review only

(Figure 1) There was also a slight increase in the rate of new diagnoses of HTN, hypercholesterolaemia and diabetes between 12 weeks and six months post NHSHC. (**Figure 1**) After that period the rate of new diagnoses plateaued and remained stable through the study. (**Figure 1**) In contrast to HTN, hypercholesterolaemia and diabetes, the rate of new diagnoses of atrial fibrillation was similar through the study duration.

There was a discrepancy between the proportion of diagnoses coded in medical records and observation of abnormal clinical and laboratory measurements. (**Table 2**) Within 12 weeks from recruitment to the NHSHC, abnormal BP measurements (SBP≥140mmHg and/or DBP≥90mmHg) were recorded for 27% (n=7,516) individuals whilst coded diagnosis of HTN was recorded for only 2.3% (n=628). Similar pattern was observed for the clinical diagnosis of hypercholesterolaemia, obesity, and CKD. Particularly, 58.7% (n=16,379) and 2% (n=547) individuals were found to have TCh≥5mmol/l and TCh≥7.5mmol/l, respectively, whilst only 70 (0.25%) individuals received a coded diagnosis of hypercholesterolaemia. Coded diagnoses of obesity and CKD were recorded for 0.5% (n=144) and 0.07% (n=19) patients, respectively. However, within the same period values of BMI meeting criteria for obesity (≥30 kg/m^2 and ≥27.5 kg/m^2 for Asian participants) and eGFR (<60 ml/min/1.73m2) were recorded for 26.2% (n=7,315) and 0.9% (n=174) patients, respectively. (**Table 2**)

Table 2 Comparison of coded diagnoses to rates of abnormal results with 12 weeks of the NHS Health Check

Diagnoses made within 12 weeks from NHSHC	Counts (%)
Clinical diagnosis of HTN	628 (2.3)
Abnormal BP reading (SBP≥140mmHg and/or DBP≥90mmHg)	7,516 (27)
Clinical diagnosis of hypercholesterolaemia	70 (0.25)
High total cholesterol (TCh ≥5mmol/l)	16,379 (58.7)
Very high total cholesterol (TCh ≥7.5mmol/l)	547 (2)
Clinical diagnosis of diabetes mellitus	248 (0.9)
Clinical diagnosis of obesity	144 (0.5)
BMI meeting criteria for obesity (≥30 kg/m2 and ≥27.5 kg/m2 for Asian participants)	7,315 (26.2)
Clinical diagnosis of CKD	19 (0.07)
Abnormal eGFR (<60ml/min/1.73m2)	174 (0.9)

For peer review only - http://bmjopen.bmj.com/site/about/guidelines.xhtml

NHSHS – NHS Health Check, HTN – hypertension, BP – Blood pressure, SBP – Systolic blood pressure, DBP – Diastolic blood pressure TCh – total cholesterol, BMI – body mass index, CKD – chronic kidney disease, eGFR – estimated glomerular filtration rate.

Medical Prescriptions and clinical interventions

Within the first 12 weeks following the NHSHC visit, 5.4% (1,510) of all attendees were started on antihypertensive medications, which increased to 8.4% (2,337) at 18 months. Within the first 12 weeks from the NHSHC visit antihypertensive medications were prescribed for 3.6%, 10.0% and 21.2% of participants with low, high and very high CVD risk, respectively and 9.7%, 26.7% and 61.2% for grade one, two and three HTN, respectively. At 18 months, these proportions increased to 6.4%, 14.8% and 26.2%, for the three CVD risk groups, and to 15.4%, 36.9% and 63.1% for the three HTN groups, respectively. At 18 months 4.5%, 13.5% and 28.8% of GENVASC participants, meeting criteria for grade one, grade two and grade three HTN, respectively were prescribed more than one group of antihypertensive medications. The time from the NHSHC to onset of antihypertensive medication showed big discrepancies, however there was a strong trend for shorter time to pharmacological treatment with higher category of HTN. In particular, median time from NHSHC to first prescription was reduced from 23 weeks for patients with grade one HTN, to 12 weeks for those with grade two HTN and 6 weeks for those with grade three HTN.

Similar trends were observed for the prescription of lipid lowering medications. Statins were prescribed to 5.4% (1,514) of all attendees and the proportion gradually increased to 7.1% (1,984) at 18 months. The proportion of patients prescribed statins increased with the severity of CVD risk, whilst the median time to statin prescription was decreased as the severity of CVD risk was increased. Specifically, within the first 12 weeks following the NHSHC, statins were prescribed to 1.8%, 14.2% and 34.2% of individuals in the low, high and very high CVD risk groups respectively, reaching 2.8%, 18% and 39.2% at 18 months. The median time from the NHSHC to statin prescription was 29 weeks for the low CVD risk group, reduced to seven and four weeks for the high and very high CVD risk groups, respectively.

For peer review only - http://bmjopen.bmj.com/site/about/guidelines.xhtml

Within the first 12 weeks following the NHSHC, 5.3% of participants with high TCh and 24.5% of participants with very high TCh were started on statins, reaching 7.4% and 33%, at 18 months, respectively.

Frequency of follow-up

The frequency of follow-up visits was mainly associated with the severity of the abnormality detected during the NHSHC.

Over 18 months, patients, with grade one, two and three hypertension, as assessed during NHSHC visit, had a median of 4 (95% CI 2-9), 4 (95% CI 2-11), and 6 (95% CI 2-12) measurements of BP, respectively. Over 18 months of follow-up, at least a single repeated measurement of BP was performed in 41% of patients with normal, 58% with grade one, 81% with grade two and 88.5% of patients with grade three HTN, respectively.

Over 18 months of follow up repeated measurements of TCh were performed in 20%, 28% and 57% of patients with normal, high and very high TCh, respectively.

In contrast to the repeated measurements of BP and TCh, repeated measurements of body weight were done less frequently, with around 1 in 4 participants in total having a record of repeated weight measurements over the 18 months. At least a single repeated weight measurement was recorded for 17% of overweight and 25% of obese participants over 18 months of follow up. There was a strong association between the overall cardiovascular risk and repeated measurements of BMI. At least on repeated measurement of BMI was performed for 24%, 30% and 37% of participants with low, high and very high CVD risk, respectively.

In addition to association with CVD risk there was an association between prescription of statins and anti-hypertensive medications and follow up. Among patients with evidence of follow up antihypertensive medications and statins were prescribed to 24% and 29.8% in comparison to 2.2% and 5.7 % of patients with no evidence of follow up. (Table 4)

Change in the levels of CVD risk factors (first repeated measurement after 12 months from the NHSHC)

For peer review only - http://bmjopen.bmj.com/site/about/guidelines.xhtml

The magnitude of changes in the CVD risk factors during the follow-up period were relevant to the attendant's initial CVD risk severity group, allocated during the NHSHC visit, and were more evident in participants in the very high risk group.

At follow up participants in the low CVD risk category, had on average higher SBP and DBP by 3.6 (3.1; 3.9) and 1.2 (1.0; 1.6) mmHg, respectively (average time to observation 74+/-15 weeks), and lower median eGFR by 5 (-5.6; -4.3) ml/min/1.73m² (average time to observation 75+/-15 weeks). There was a minor reduction in the TCh levels by 0.14 (-0.16; -0.10) mmol/l, (average time to repeated measurement 76+/-15 weeks). (**Table 3, low CVD group**)

For participants in the high CVD risk group, there was no significant change in the SBP. DBP decreased by on average 0.9 (-1.4; -0.40) mmHg (average time to the follow up measurement 73+/-15 weeks), whilst total and non-HDL cholesterol were decreased by 0.45 (-0.51; -0.39) and 0.47 (-0.55; -0.39) mmol/l, respectively (average time to repeated measurement 75+/-15 weeks). The median eGFR was lower by 3 (-4.4; -1.6) ml/min/1.73m² (average time to repeated measurement 74+/-15 weeks). There was no significant change in the BMI at follow up. (**Table 3, high CVD group**)

Participants in the very high CVD risk group, evidenced reduction, on average, in the SBP and DBP levels by 7.5 (-9.4; -5.7) and 5 (-6.0; -3.9) mmHg, respectively (average time to repeated measurement 71+/-8 weeks) and in median eGFR by 3 (-5.0; -0.8) ml/min/1.73m^2 (average time to repeated measurement 74 +/-15 weeks). There were also clinically significant reductions in TCh and non-HDL cholesterol by 0.79 (-0.91; -0.69) mmol/l, -0.68 (-0.83; -0.53) mmol/l, respectively (average time to repeated measurement 74+/-15 weeks). The BMI reduced on average by -0.3 (-0.52; -0.11) kg/m^2, respectively (average time to a repeated measurement 73+/- 15 weeks). **(Table 3, very high CVD group)**

Table 3. Comparison of clinical characteristic of participants between NHSHC and follow-up.

Parameters	NHSHC	Follow-up	Delta (FU-HC)	P

For peer review only - http://bmjopen.bmj.com/site/about/guidelines.xhtml

		(12-18 months)	(95% CI)	
**Low CVD risk (QRISK2<10)**				
Obesity, n=3,747				
Body mass index, kg/m^2	28.0 (5.9)	28.0 (5.9)	0.006 (-0.07; 0.05)	0.83
Blood pressure, n=6,785				
Systolic, mmHg	125.5 (15.6)	129.1 (16.3)	3.6 (3.1;3.9)	<0.001
Diastolic, mmHg	78.7 (10.2)	79.9 (10.4)	1.2 (1.0; 1.6)	<0.001
Cholesterol, n=3,233				
Total cholesterol, mmol/l	5.4 (1.0)	5.3 (0.98)	-0.14 (-0.16; -0.10)	<0.001
Non-HDL-cholesterol, n=1,339				
Non-HDL-cholesterol, mmol/l	3.9 (1.0)	3.7 (0.98)	-0.12 (-0.16; -0.08)	<0.001
Median eGFR, n=2,551				
eGFR, ml/min/1.73m²	86 (85; 87)	81 (75; 86)	-5 (-5.6; -4.3)	<0.0001
**High CVD risk (QRISK2 10-20)**				
Obesity, n=1214				
Body mass index, kg/m^2	27.6 (5.4)	27.6 (5.7)	-0.08 (-0.20; 0.06)	0.27
Blood pressure, n=2046				
Systolic blood pressure, mmHg	136.7 (16.5)	136.4 (16.6)	-0.3 (-1.1; 0.53)	0.5
Diastolic blood pressure, mmHg	81.1 (10.8)	80.2 (10.2)	-0.9 (-1.4; -0.40)	<0.001
Cholesterol, n=1,339				
Total cholesterol, mmol/l	5.6 (1.1)	5.1 (1.1)	-0.45 (-0.51; -0.39)	<0.001
Non-HDL-cholesterol, n=583				
Non HDL-cholesterol, mmol/l	4.0 (1.0)	3.6 (1.1)	-0.47 (-0.55; -0.39)	<0.001
Median eGFR, n=1,129				
eGFR, ml/min/1.73m²	82 (78; 81)	79 (78; 80)	-3 (-4.4; -1.6)	<0.001
**Very high CVD risk (QRISK2>20)**				
Obesity, n=354				
Body mass index, kg/m^2	28.7 (6.1)	28.4 (6.0)	-0.3 (-0.52; -0.11)	0.002
Blood pressure, n=571				
Systolic blood pressure, mmHg	146.2 (20.8)	138.7 (16.3)	-7.5 (-9.4; -5.7)	<0.001
Diastolic blood pressure, mmHg	84.8 (12.5)	79.8 (10.4)	-5 (-6.0; -3.9)	<0.001
Cholesterol, n=419				
Total cholesterol, mmol/l	5.6 (1.1)	4.8 (1.2)	-0.79 (-0.91; -0.69)	<0.001
Non-HDL-cholesterol, n=174				
Non HDL-cholesterol, mmol/l	4.1 (1.0)	3.4 (1.1)	-0.68 (-0.83; -0.53)	<0.001
Median eGFR, n=303				
eGFR, ml/min/1.73m²	81 (79; 83)	78 (76; 80)	-3 (-5.0; -0.8)	0.006

Results are shown as mean (SD) unless otherwise indicated. Differences in mean values or proportions between the follow-up and the NHSHC, along with the relevant 95% Confidence intervals, are also shown. P-values derived from the paired t-test for the continuous variables and the McNemar test for the nominal paired variables. Blood pressure (BP) was defined as normal if systolic blood pressure (SBP) <140mmHg and/or diastolic blood pressure (DBP) <90mmHg; Total cholesterol (TCh) was defined as normal for values <5.0mmol/l; Non-high density lipoprotein (non-HDL) was defined as

For peer review only - http://bmjopen.bmj.com/site/about/guidelines.xhtml

normal if values were <3.8mmol/l; Kidney function was considered normal if eGFR≥60ml/min/1.73xm^2; Body weight was considered as healthy body weight if BMI <25kg/m^2 (<23 kg/m^2 for South Asians).

Analogous to the gradient of CVD risk, the magnitude of the changes in the individual risk factors levels varied according the severity of the abnormality observed for that risk factor, during the NHSHC. Overall, individuals with BP above 140/90 mmHg, as measured during the NHSHC, achieved an average reduction of 7 (-7.7; -6.3) mmHg of SBP and 4.6 (-5.1; -4.2) mmHg of DBP (average time to repeated observation 73+/-15 weeks). For grade one, grade two and grade three HTN patients, SBP decreased on average by 2.9 (-3.7; -2.3), 15.7 (-17.5; -14.1) and 33.4 (-37.7; -29.4;) mmHg, respectively. **(Figure 2, top panel)**

A similar pattern, was observed for the TCh levels, with an average reduction of 0.38 (-0.41; -0.34) mmol/l and 1.71 (-1.94; -1.48) mmol/l, respectively, for patients with high and very high TCh (average time to repeated measurement 75+/-15 weeks) **(Figure 2, middle panel)**

There was a small reduction in the BMI with overweight patients losing approximately 0.08 (-0.15; -0.007) kg/m^2 and obese patients losing 0.27 (-0.37; -0.12) kg/m^2 (average time to repeated observation 75+/-15 weeks). **(Figure 2, bottom panel)**

<u>Smoking cessation</u>

During the NHSHC, 16% (3,813; 19% males, 14% females) were recorded as current smokers. Of the 3,813 current smokers, 56% (2,133; 53% males, 47% females) had a record of smoking cessation advice documented during or within 30 days of the NHSHC. This proportion increased to 60% (2,275) at 18 months, whilst 14% of participants had multiple records of advice for smoking cessation. At 18 months 451(12%) participants quit smoking, according to the GP records.

<u>Individuals with no record of follow-up</u>

For peer review only

For peer review only - http://bmjopen.bmj.com/site/about/guidelines.xhtml

The demographic and clinical characteristics of high and very high CVD risk patients with unrecorded follow-up are presented in **Table 4**. Approximately 1 in 3 patients with high or very high CVD risk had no recorded evidence of a clinical follow-up in the 18-month period following the NHSHC. Lack of clinical follow-up was associated with male sex and white ethnic background and lower index of social deprivation. (**Table 4**) Participants without evidence of follow-up were more likely to be ex-smokers, had, at the time of the NHSHC, on average lower values of BMI, SBP and DBP than individuals who had record of follow-up, albeit the differences were small. (**Table 4**) Results from the logistic regression analysis showed that other than white ethnic background (OR (95% CI): (1.79 (1.33; 2.17), p<0.001), younger age (-1.02 (-1.06; -1.001), p=0.004), greater SBP (1.02 (1.01; 1.03), p<0.001), greater BMI (1.02 (1.01; 1.04), p=0.001) and higher Townsend score (more deprived) (1.04 (1.01; 1.06)), were independently associated with presence of follow-up record.

Table 4. Demographic and clinical characteristics of participants with high and very high CVD risk who did not have record of follow-up.

	Follow up	No follow up	Delta (N-Y) (95% CI)	P
**High &very high CVD risk, n (%) (QRISK2 ≥10)**	4,217 (67.5)	2,035 (32.5)		
<u>*Demographic*</u>				
Male sex, n (%)	2,738 (64.9)	1,378 (67.7)	2.8 (0.3; 5.2)	0.0290
White ethnic background, n (%)	3,615 (85.7)	1,850 (90.9)	5.2 (3.5; 6.8)	<0.0001
Age, years (Median, CI)	64.2 (63.9-64.4)	64.6 (64.4-64.9)	0.47 (0.11; 0.83)	0.0104
Quintiles of deprivation, n (%)				
1st quintile	1240 (29.9)	656 (32.8)	-2.9 (-5.4; -0.4)	0.0229
2nd quintile	875 (21.1)	483 (24.1)	-3.0 (-5.3; 0.8)	0.0077
3rd quintile	707(17.0)	305 (15.2)	1.8 (-0.1: 3.7)	0.0809
4th quintile	490 (11.8)	236 (11.8)	-	1

For peer review only - http://bmjopen.bmj.com/site/about/guidelines.xhtml

5th quintile (most deprived)	841 (20.2)	323 (16.1)	4.1 (2.0: 6.2)	0.0001
<u>*Clinical*</u>				
Current smokers, n (%)	781 (18.5)	359 (17.6)	-0.9 (-0.03; 0.11)	0.3876
Ex-smokers, n (%)	1,176 (27.9)	501 (24.6)	-3.3 (-5.6; -0.9)	0.0058
BMI, kg/m2	27.9 (5.2)	26.9 (4.7)	-1.0 (-1.0; -0.66)	<0.0001
Systolic blood pressure, mmHg	139.3 (17.8)	132.6 (13.5)	-6.7 (-7.0; -5.9)	<0.0001
Diastolic blood pressure, mmHg	82.3 (11.2)	78.7 (8.9)	-3.6 (-4.0; 3.1)	<0.0001
Antihypertensive medications, n (%)	1,012 (24.0)	44 (2.2)	-21.8 (-23.2; -20)	<0.0001
Total cholesterol, mmol/l	5.6 (1.1)	5.5 (1.0)	-0.1 (-0.1; -0.02)	0.0079
Non-HDL cholesterol, mmol/l	4.0 (1.0)	3.9 (0.9)	-0.1 (-0.1, 0.01)	0.0622
Statins, n (%)	1,257 (29.8)	116 (5.7)	-24.1 (-25.8-; -22.4)	<0.0001

Results are mean (SD) unless otherwise stated. Blood pressure (BP) was defined as normal if systolic blood pressure (SBP) <140mmHg and/or diastolic blood pressure (DBP) <90mmHg; Total cholesterol (TCh) was defined as normal for values <5.0mmol/l; Non-high density lipoprotein (non-HDL) was defined as normal if values were <3.8mmol/l; Body weight was considered as healthy body weight if BMI <25kg/m^2 (<23 kg/m^2 for South Asians).

Discussion

We present results evaluating the clinical effectiveness of the NHSHC in identifying, treating and monitoring individuals at high CVD risk. Our study highlights novel findings which may influence implementation of strategies for improving effectiveness of the programme.

A key finding from our study is the characterisation of high and very high CVD risk patients (QRISK2≥10), who had attended the NHSHC, but have not had any record of clinical follow-up over 18 months and have not received any treatment. This is a considerably large sub-population of patients and our study showed that this group has specific social, demographic and clinical characteristics. It is also the group of patients, where preventive interventions are likely to bring the largest clinical benefits and be most cost effective. Similar observations were made for patients with very abnormal values of isolated risk factors - such as blood pressure and cholesterol. These subgroups of participants have a very high CVD

For peer review only - http://bmjopen.bmj.com/site/about/guidelines.xhtml

For peer review only

risk, although it may not be accurately estimated by QRISK2.[13] This group of patients may need multidisciplinary approach utilising specialist services including genetic testing and cascade screening, which cannot be provided in GP practices.

The second important finding relates to the level of control of risk factors. Despite the substantial improvements in both BP and TCh, their values at 18 months remained high. (**Figure 2**) This indicates that even when patients were started on treatment, there does not seem to be an imperative to ensure that the risk factors are well controlled. This may be due to several factors including high workload of GP practices and lack of clear definition of primary prevention treatment targets. It is also likely that it reflects the way primary care is incentivised. GP practices receive a payment from NHS once a new case is identified or a new treatment is being initiated, rather than receiving a payment once treatment goals have been achieved. [21] Our study suggests that this mechanism may be insufficient. Our data also showed lack of adherence to NICE recommendation, which advocates re-examination of lipid profile three months after prescribing statins.[14] Lack of repeated cholesterol measurements may be one of the factors significantly reducing uptake and continuation of statin treatment.[22]

Our findings are consistent with previous research indicating that attendance to NHSHC is associated with increased detection of CVD and its risk factors and prescription of therapy.[8, 10, 23] It also confirms the previous findings that the magnitude of the improvement in risk factors is relevant to the severity of the disease.[8, 10] Based on the example of high blood pressure management, our study provides strong evidence of the clinical effectiveness of NHSHC as a public programme aimed at reduction of CVD risk. However, the general perception of NHSC should be changed. NHSHC has to be recognised as a sequence of events starting with detection of increased CVD risk during the NHSHC, followed by general advice and prescription of therapy, and follow-up monitoring. Then a reassessment is required to decide if further changes to therapy are needed.

Our data indicate that the problem of patients with increased CVD risk (QRISK2≥10) not subjected to follow-up has not been adequately explored. A previous survey performed in general practices in London in the first year after implementation of the NHSHC identified

For peer review only - http://bmjopen.bmj.com/site/about/guidelines.xhtml

problems with implementation.[24] The identified problems included non-prescribing of statins to high risk individuals, reluctance to refer to external services and variable patterns of organising clinical follow-up with only around 50% of practices organising the recommended annual recall.[24] Forester et al. (2015) also reported that fewer than 52% of patients had repeated monitoring of CVD risk factors over the 15 month period of observation.[8] Several studies reported lower than expected prescriptions of statins to patients diagnosed with elevated TCh and/or high CVD risk during NHSHC.[8-10]

Lower than projected uptake of the NHSHC has been a concern as the major factor influencing cost-effectiveness of the programme.[25] Recent data indicate a steady increase in the uptake now reaching a satisfactory level of 52%.[16, 26] Our study indicates that overall effectiveness of the NHSHC can be improved by optimising delivery of interventions reducing the CVD risk.

Limitations of the study

Our analysis has several limitations. Not all patients attending the NHSHC and invited to the GENVASC study took part and the exact proportion (uptake) is not known. It is possible that the patients who participated in the study were more proactive in attending follow up appointments and undertaking medical and lifestyle interventions than the general population of attenders to the NHSHC. Our analysis was performed in a population with higher than the average for UK proportion of Asian participants. The performed analysis focused on CVD risk factors recorded in the GPs records but no data were available regarding lifestyle changes (e.g. on physical exercise, diet or alcohol consumption). Furthermore, we do not have data on prescription of anti-obesity drugs, or referral to weight loss, alcohol, or diabetes prevention services.

Acknowledgements: The research team would like to express gratitude to all participant of the GENVASC study. We would also like to thank all healthcare and other professionals involved in performing NHS Health Check as well as research related tasks.

For peer review only - http://bmjopen.bmj.com/site/about/guidelines.xhtml

Funding: The GENVASC study is funded by the NIHR Leicester Biomedical Research Centre (Grant No. BRC-1215-20010). RD is funded by NIHR – grant number NA. KK is supported by the National Institute for Health Research (NIHR) Applied Research Collaboration East Midlands (ARC EM) and the NIHR Leicester Biomedical Research Centre (BRC) – grant number NA.

Conflicts of interest: KK was a national advisor for the NHS Health Checks Programme.

Author contributions:

RD - designed the project, performed analysis and is responsible for the overall contents of the manuscript.

VB - designed the project, performed analysis, supervised the statistical analysis and is responsible for the overall contents of the manuscript.

RP- designed the project, performed analysis and is responsible for the overall contents of the manuscript.

NJS - designed the project, performed analysis and is responsible for the overall contents of the manuscript.

DL - took part in collection and analysis of data, was involved in managing of the research databases and download of data, reviewed the content and contributed to the final manuscript.

EB - took part in collection and analysis of data, was involved in overall management of the study, reviewed the content and contributed to the final manuscript.

CG - took part in collection and analysis of data, was involved in managing of the research databases and download of data, reviewed the content and contributed to the final manuscript.

RB - took part in collection and analysis of data, was involved in managing of the research databases and download of data, reviewed the content and contributed to the final manuscript.

SS - took part in collection and analysis of data, was involved in managing of the research databases and download of data, reviewed the content and contributed to the final manuscript.

For peer review only

For peer review only - http://bmjopen.bmj.com/site/about/guidelines.xhtml

SK - took part in collection and analysis of data, reviewed the content and contributed to the final manuscript.

MN - took part in collection and analysis of data, was involved in and overlooked clinical governance of the project, reviewed the content and contributed to the final manuscript.

AM - took part in collection and analysis of data, was involved in overall management of the study, reviewed the content and contributed to the final manuscript.

AK - took part in collection and analysis of data, reviewed the content and contributed to the final manuscript.

AZ - took part in collection and analysis of data, reviewed the content and contributed to the final manuscript.

AC - took part in collection and analysis of data, reviewed the content and contributed to the final manuscript.

AH - took part in collection and analysis of data, reviewed the content and contributed to the final manuscript.

KK - took part in collection and analysis of data, reviewed the content and contributed to the final manuscript.

NJ - took part in collection and analysis of data, reviewed the content and contributed to the final manuscript.

ML - took part in collection and analysis of data, reviewed the content and contributed to the final manuscript.

AF - took part in collection and analysis of data, reviewed the content and contributed to the final manuscript.

Data availability: Data available on request from authors subject to privacy/ethical restrictions.

For peer review only - http://bmjopen.bmj.com/site/about/guidelines.xhtml

Research Ethics Approval: Human Participants The study was approved by the East Midlands - Derby Research Ethics Committee (approval number 12/EM/0208). All study subjects gave informed consent for participation.

Figure Legends:

Figure 1: Weekly incidence of CVD risk factors in relation to NHS Health Check.,

Figure 2: Magnitude of changes in the individual risk factors during follow up.

For peer review only

For peer review only - http://bmjopen.bmj.com/site/about/guidelines.xhtml

References

1. Murray CJ, Richards MA, Newton JN, Fenton KA, Anderson HR, Atkinson C, Bennett D, Bernabe E, Blencowe H, Bourne R, Braithwaite T, Brayne C, Bruce NG, Brugha TS, Burney P, Dherani M, Dolk H, Edmond K, Ezzati M, Flaxman AD, Fleming TD, Freedman G, Gunnell D, Hay RJ, Hutchings SJ, Ohno SL, Lozano R, Lyons RA, Marcenes W, Naghavi M, Newton CR, Pearce N, Pope D, Rushton L, Salomon JA, Shibuya K, Vos T, Wang H, Williams HC, Woolf AD, Lopez AD, Davis A. UK health performance: findings of the Global Burden of Disease Study 2010. Lancet 2013;**381**(9871):997-1020.
2. Roth GA, Johnson C, Abajobir A, Abd-Allah F, Abera SF, Abyu G, Ahmed M, Aksut B, Alam T, Alam K, Alla F, Alvis-Guzman N, Amrock S, Ansari H, Arnlov J, Asayesh H, Atey TM, Avila-Burgos L, Awasthi A, Banerjee A, Barac A, Barnighausen T, Barregard L, Bedi N, Belay Ketema E, Bennett D, Berhe G, Bhutta Z, Bitew S, Carapetis J, Carrero JJ, Malta DC, Castaneda-Orjuela CA, Castillo-Rivas J, Catala-Lopez F, Choi JY, Christensen H, Cirillo M, Cooper L, Jr., Criqui M, Cundiff D, Damasceno A, Dandona L, Dandona R, Davletov K, Dharmaratne S, Dorairaj P, Dubey M, Ehrenkranz R, El Sayed Zaki M, Faraon EJA, Esteghamati A, Farid T, Farvid M, Feigin V, Ding EL, Fowkes G, Gebrehiwot T, Gillum R, Gold A, Gona P, Gupta R, Habtewold TD, Hafezi-Nejad N, Hailu T, Hailu GB, Hankey G, Hassen HY, Abate KH, Havmoeller R, Hay SI, Horino M, Hotez PJ, Jacobsen K, James S, Javanbakht M, Jeemon P, John D, Jonas J, Kalkonde Y, Karimkhani C, Kasaeian A, Khader Y, Khan A, Khang YH, Khera S, Khoja AT, Khubchandani J, Kim D, Kolte D, Kosen S, Krohn KJ, Kumar GA, Kwan GF, Lal DK, Larsson A, Linn S, Lopez A, Lotufo PA, El Razek HMA, Malekzadeh R, Mazidi M, Meier T, Meles KG, Mensah G, Meretoja A, Mezgebe H, Miller T, Mirrakhimov E, Mohammed S, Moran AE, Musa KI, Narula J, Neal B, Ngalesoni F, Nguyen G, Obermeyer CM, Owolabi M, Patton G, Pedro J, Qato D, Qorbani M, Rahimi K, Rai RK, Rawaf S, Ribeiro A, Safiri S, Salomon JA, Santos I, Santric Milicevic M, Sartorius B, Schutte A, Sepanlou S, Shaikh MA, Shin MJ, Shishehbor M, Shore H, Silva DAS, Sobngwi E, Stranges S, Swaminathan S, Tabares-Seisdedos R, Tadele Atnafu N, Tesfay F, Thakur JS, Thrift A, Topor-Madry R, Truelsen T, Tyrovolas S, Ukwaja KN, Uthman O, Vasankari T, Vlassov V, Vollset SE, Wakayo T, Watkins D, Weintraub R, Werdecker A, Westerman R, Wiysonge CS, Wolfe C, Workicho A, Xu G, Yano Y, Yip P, Yonemoto N, Younis M, Yu C, Vos T, Naghavi M, Murray C. Global, Regional, and National Burden of Cardiovascular Diseases for 10 Causes, 1990 to 2015. J Am Coll Cardiol 2017;**70**(1):1-25.
3. Health Do. Putting prevention first: Vascular checks risk assessment and management - impact assessment. 2008.
4. Health Do. Economic Modelling for Vascular Checks 2008.
5. Krogsbøll LT, Jørgensen KJ, Gøtzsche PC. Universal health checks should be abandoned. BMJ : British Medical Journal 2013;**347**:f5227.
6. Soljak M, Majeed A, Millett C. Response to Krogsbøll and colleagues: NHS health checks or government by randomised controlled trial? BMJ : British Medical Journal 2013;**347**:f5984.
7. Chang KC, Lee JT, Vamos EP, Soljak M, Johnston D, Khunti K, Majeed A, Millett C. Impact of the National Health Service Health Check on cardiovascular disease risk: a difference-in-differences matching analysis. CMAJ 2016;**188**(10):E228-E238.
8. Forster AS, Dodhia H, Booth H, Dregan A, Fuller F, Miller J, Burgess C, McDermott L, Gulliford MC. Estimating the yield of NHS Health Checks in England: a population-based cohort study. J Public Health (Oxf) 2015;**37**(2):234-40.
9. Caley M, Chohan P, Hooper J, Wright N. The impact of NHS Health Checks on the prevalence of disease in general practices: a controlled study. Br J Gen Pract 2014;**64**(625):e516-21.
10. Kennedy O, Su F, Pears R, Walmsley E, Roderick P. Evaluating the effectiveness of the NHS Health Check programme in South England: a quasi-randomised controlled trial. BMJ Open 2019;**9**(9):e029420.
11. Cochrane T, Davey R, Iqbal Z, Gidlow C, Kumar J, Chambers R, Mawby Y. NHS health checks through general practice: randomised trial of population cardiovascular risk reduction. BMC Public Health 2012;**12**:944.
12. England PH. NHS Health Check Best practice guidance. 2019.

For peer review only - http://bmjopen.bmj.com/site/about/guidelines.xhtml

13. Hippisley-Cox J, Coupland C, Vinogradova Y, Robson J, Minhas R, Sheikh A, Brindle P. Predicting cardiovascular risk in England and Wales: prospective derivation and validation of QRISK2. BMJ 2008;**336**(7659):1475-82.

14. (NICE) NIfHaCE. Cardiovascular disease: risk assessment and reduction, including lipid modification. Clinical guideline [CG181]. 2014.

15. Centere NLBR. https://www2.le.ac.uk/projects/bru/our-research/research-themes/genetics-and-biomarkers/genvasc.

16. Patel R, Barnard S, Thompson K, Lagord C, Clegg E, Worrall R, Evans T, Carter S, Flowers J, Roberts D, Nuttall M, Samani NJ, Robson J, Kearney M, Deanfield J, Waterall J. Evaluation of the uptake and delivery of the NHS Health Check programme in England, using primary care data from 9.5 million people: a cross-sectional study. BMJ Open 2020;**10**(11):e042963.

17. Mancia G, Fagard R, Narkiewicz K, Redon J, Zanchetti A, Bohm M, Christiaens T, Cifkova R, De Backer G, Dominiczak A, Galderisi M, Grobbee DE, Jaarsma T, Kirchhof P, Kjeldsen SE, Laurent S, Manolis AJ, Nilsson PM, Ruilope LM, Schmieder RE, Sirnes PA, Sleight P, Viigimaa M, Waeber B, Zannad F, Redon J, Dominiczak A, Narkiewicz K, Nilsson PM, Burnier M, Viigimaa M, Ambrosioni E, Caufield M, Coca A, Olsen MH, Schmieder RE, Tsioufis C, van de Borne P, Zamorano JL, Achenbach S, Baumgartner H, Bax JJ, Bueno H, Dean V, Deaton C, Erol C, Fagard R, Ferrari R, Hasdai D, Hoes AW, Kirchhof P, Knuuti J, Kolh P, Lancellotti P, Linhart A, Nihoyannopoulos P, Piepoli MF, Ponikowski P, Sirnes PA, Tamargo JL, Tendera M, Torbicki A, Wijns W, Windecker S, Clement DL, Coca A, Gillebert TC, Tendera M, Rosei EA, Ambrosioni E, Anker SD, Bauersachs J, Hitij JB, Caulfield M, De Buyzere M, De Geest S, Derumeaux GA, Erdine S, Farsang C, Funck-Brentano C, Gerc V, Germano G, Gielen S, Haller H, Hoes AW, Jordan J, Kahan T, Komajda M, Lovic D, Mahrholdt H, Olsen MH, Ostergren J, Parati G, Perk J, Polonia J, Popescu BA, Reiner Z, Ryden L, Sirenko Y, Stanton A, Struijker-Boudier H, Tsioufis C, van de Borne P, Vlachopoulos C, Volpe M, Wood DA. 2013 ESH/ESC guidelines for the management of arterial hypertension: the Task Force for the Management of Arterial Hypertension of the European Society of Hypertension (ESH) and of the European Society of Cardiology (ESC). Eur Heart J 2013;**34**(28):2159-219.

18. Piepoli MF, Hoes AW, Agewall S, Albus C, Brotons C, Catapano AL, Cooney MT, Corra U, Cosyns B, Deaton C, Graham I, Hall MS, Hobbs FDR, Lochen ML, Lollgen H, Marques-Vidal P, Perk J, Prescott E, Redon J, Richter DJ, Sattar N, Smulders Y, Tiberi M, van der Worp HB, van Dis I, Verschuren WMM, Binno S, Group ESCSD. 2016 European Guidelines on cardiovascular disease prevention in clinical practice: The Sixth Joint Task Force of the European Society of Cardiology and Other Societies on Cardiovascular Disease Prevention in Clinical Practice (constituted by representatives of 10 societies and by invited experts)Developed with the special contribution of the European Association for Cardiovascular Prevention & Rehabilitation (EACPR). Eur Heart J 2016;**37**(29):2315-2381.

19. (NICE). NIfHaCE. Chronic kidney disease in adults: assessment and management. Clinical guideline [CG182]. 2014.

20. StataCorp. Stata Statistical Software: Release 16. College Station, TX: StataCorp LLC. 2019.

21. Health Do. Putting Prevention First, Vascular Check: Risk Assessment and Management. London: Department of Health; 2008.

22. Benner JS, Tierce JC, Ballantyne CM, Prasad C, Bullano MF, Willey VJ, Erbey J, Sugano DS. Follow-up lipid tests and physician visits are associated with improved adherence to statin therapy. Pharmacoeconomics 2004;**22 Suppl 3**:13-23.

23. Forster AS, Burgess C, Dodhia H, Fuller F, Miller J, McDermott L, Gulliford MC. Do health checks improve risk factor detection in primary care? Matched cohort study using electronic health records. J Public Health (Oxf) 2016;**38**(3):552-559.

24. Nicholas JM, Burgess C, Dodhia H, Miller J, Fuller F, Cajeat E, Gulliford MC. Variations in the organization and delivery of the 'NHS health check' in primary care. J Public Health (Oxf) 2013;**35**(1):85-91.

For peer review only - http://bmjopen.bmj.com/site/about/guidelines.xhtml

25. Martin A, Saunders CL, Harte E, Griffin SJ, MacLure C, Mant J, Meads C, Walter FM, Usher-Smith JA. Delivery and impact of the NHS Health Check in the first 8 years: a systematic review. Br J Gen Pract 2018;**68**(672):e449-e459.
26. Newton JN, Thompson K. NHS Health Check: national evaluation findings and implications. CMAJ 2017;**189**(4):E172.

For peer review only

For peer review only - http://bmjopen.bmj.com/site/about/guidelines.xhtml

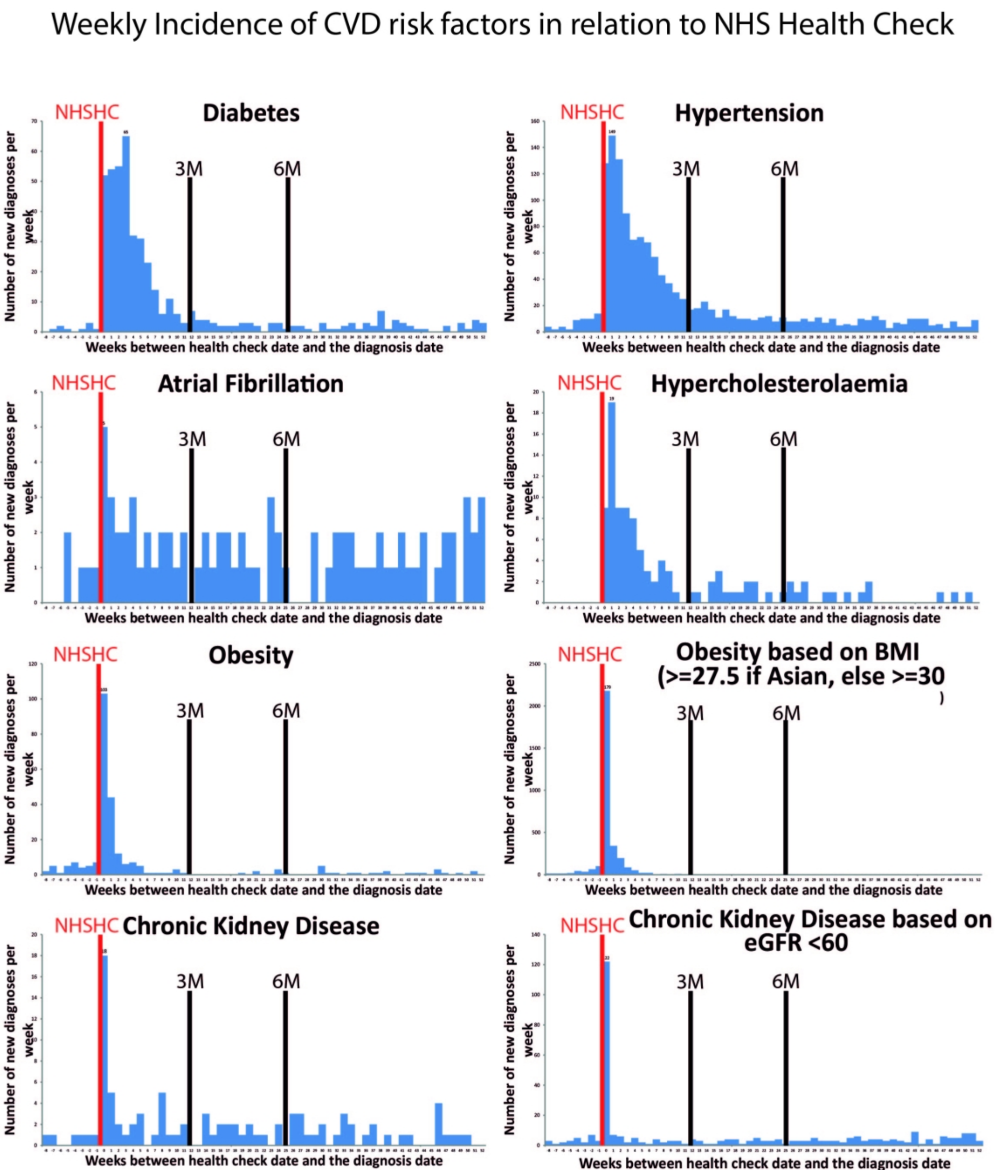

Figure 1: Weekly incidence of CVD risk factors in relation to NHS Health Check.

198x231mm (300 x 300 DPI)

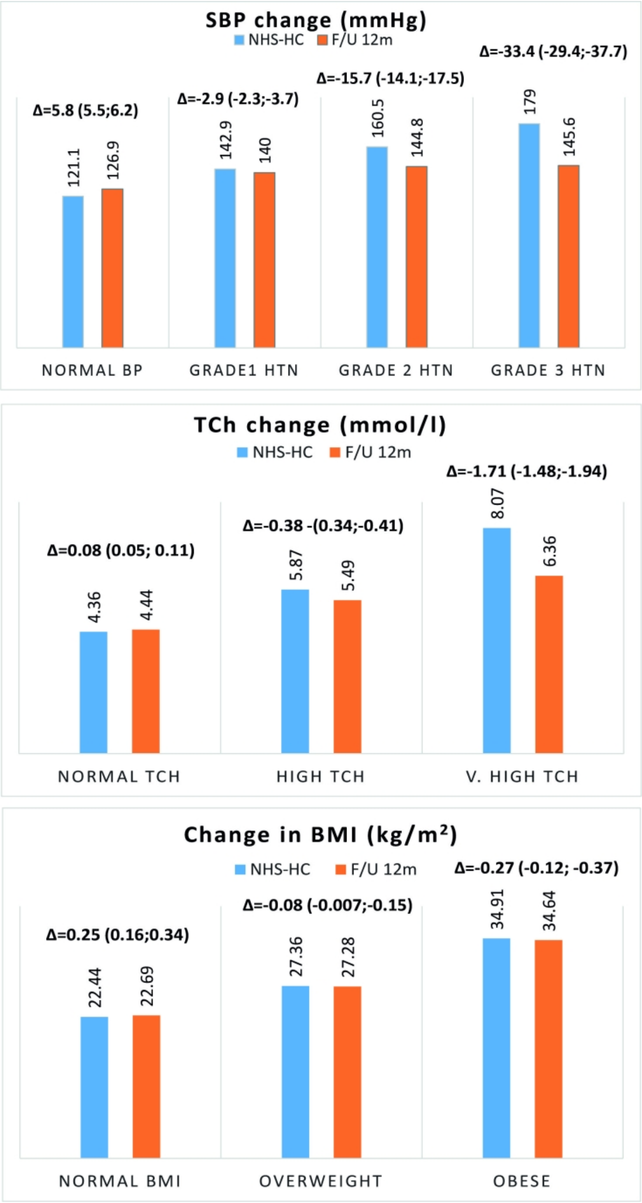

Magnitude of change in the individual risk factors

156x291mm (300 x 300 DPI)

SUPPLEMENTARY DATA

Clinical variables and measurements

BP was measured individually in GP practices using standardised oscillatory BP measuring devices after a minimum of 5-minute rest. Total Cholesterol (TC, mmol/l), High Density Lipoprotein-cholesterol (HDL-C, mmol/l) and serum creatinine (umol/l) were measured in accredited local laboratories following standard procedures. Non-HDL cholesterol (non-HDL-C, mmol/l) was calculated by subtracting value of HDL cholesterol from TC, Low Density Lipoprotein-cholesterol (LD-CL, mmol/l) was calculated using the Friedewald equation and estimated glomerular filtration rate (eGFR, ml/min/1.73xm^2) was calculated using the MDRD formula.

Townsend Deprivation Score

The Townsend Score is a composite measure of material deprivation of the population in UK. The Index was first introduced in 1987. The Townsend Score is published by the Office of National Statistics. Townsend score is calculated using four census variables for any given geographical area. These variables include: unemployment - defined as the percentage of economically active residents who are unemployed; non-car ownership measuring the number of households without a car; non-home ownership measuring the number of households renting and owning their accommodations; overcrowding – measuring whether the accommodation is suitable in size for the number of occupants. The Townsend Deprivation Scores were calculated using percentages of the weighted and normalised (where appropriate) four previously listed indicators. Townsend Deprivation Scores are usually split into quintiles to provide map visualisations of deprivation and to assess the spread of deprivation across areas.

https://statistics.ukdataservice.ac.uk/dataset/2011-uk-townsend-deprivation-scores

For peer review only - http://bmjopen.bmj.com/site/about/guidelines.xhtml

Supplementary Table 1. Participants' baseline characteristics according to CVD risk levels at the initial Health Check.

	CVD risk				
	All participants n=27,888	Low QRISK2 <10% n=21,636 (78)	High QRISK2 10%-20% n=5,090 (18)	Very high QRISK2>20% n=1,162 (4)	Data Coverage (%)
**Demographic**					
Males, n (%)	12,322 (44)	8,206 (38)	3,118 (61)	998 (86)	100.0
Age, years	51 (44; 60)	48 (43; 54)	65 (60; 69)	69 (64; 72)	100.0
Ethnicity, n (%)					99.5
White	22,677 (81)	17,212 (80)	4,477 (88)	988 (85)	
Asian	3,686 (13)	3,030 (14)	499 (9.8)	157 (13)	
Black	535 (1.9)	501 (2.3)	33 (0.7)	1 (0.1)	
Other ethnicity	990 (3.6)	893 (4.1)	81 (1.6)	16 (1.4)	
Quintiles of deprivation, n (%)					97.6
1st quintile (least deprived)	8,332 (30.6)	6,436 (30.6)	1,587 (31.7)	309 (27.1)	
2nd quintile	5,867 (21.6)	4,509 (21.4)	. 1,126 (22.4)	232 (20.3)	
3rd quintile	4,429 (16.3)	3,417 (16.2)	832 (16.6)	180 (15.8)	
4th quintile	3,415 (12.5)	2,689 (12.8)	583 (11.6)	143 (12.5)	
5th quintile (most deprived)	5,167 (19.0)	4,003 (19.0)	887 (17.7)	277 (24.3)	
**Clinical**					
Smoking					82.6
Non-smoker	13,810 (60)	11,416 (64)	2,081 (49)	313 (31)	
Ex-smoker	5,415 (23)	3,738 (21)	1,304 (31)	373 (38)	
Current smoker	3,813 (17)	2,673 (15)	834 (20)	306 (31)	
Obesity					
BMI, kg/m^2	27.9 (5.1)	27.1 (5.1)	27.5 (5.0)	28.2 (5.1)	100.0
Abnormal body weight (BMI≥25, BMI>23for Asian subjects), n (%)	18,443 (66.1)	14,099 (65.2)	3,458 (67.9)	886 (76.2)	
Blood pressure					

For peer review only - http://bmjopen.bmj.com/site/about/guidelines.xhtml

SBP, mmHg	127.6 (16.0)	124.9 (14.7)	135.6 (15.9)	143.6 (18.9)	100.0
DBP, mmHg	78.8 (10.0)	78.1 (9.7)	80.6 (10.4)	83.4 (11.7)	97.0
Elevated BP (SBP≥140mmHg and/or DBP≥90mmHg), n (%)	7,005 (25.1)	4,251 (19.7)	2,079 (40.8)	675 (58.1)	
Cholesterol					
TCh, mmol/l	5.29 (0.97)	5.22 (0.95)	5.53 (0.99)	5.53 (1.04)	81.5
Non-HDL, mmol/l	3.76 (0.96)	3.67 (0.94)	3.98 (0.96)	4.17 (1.00)	44.0
Elevated TCh (TCh≥5mmol/l), n (%)	14,140 (62.2)	10,449 (59.3)	3,016 (71.9)	675 (72.4)	
eGFR					
eGFR, ml/min/1.73m^2	90 (83, 90)	90 (85; 90)	90 (79; 90)	87 (78; 90)	73.5
Abnormal eGFR (eGFR<60 ml/min/1.73m^2), n (%)	174 (0.9)	94 (0.6)	60 (1.6)	20 (2.3)	

Results are mean (SD) or median (1st quartile; 3rd quartile) unless otherwise stated; Data coverage refers to the proportion of subjects with available data. **Abbreviations:** BMI-body mass index; SBP-systolic blood pressure; DBP-diastolic blood pressure; TCh-total cholesterol; Quintiles of deprivation were calculated using the Townsend Index of Deprivation 2019.[27] For the calculation of Qrisk2, Townsend index of deprivation was substituted with the average value of the index (i.e. "0") in participants with missing data.

For peer review only - http://bmjopen.bmj.com/site/about/guidelines.xhtml

Supplementary Figure 1. Geographical distribution of recruitment to the GENVASC study

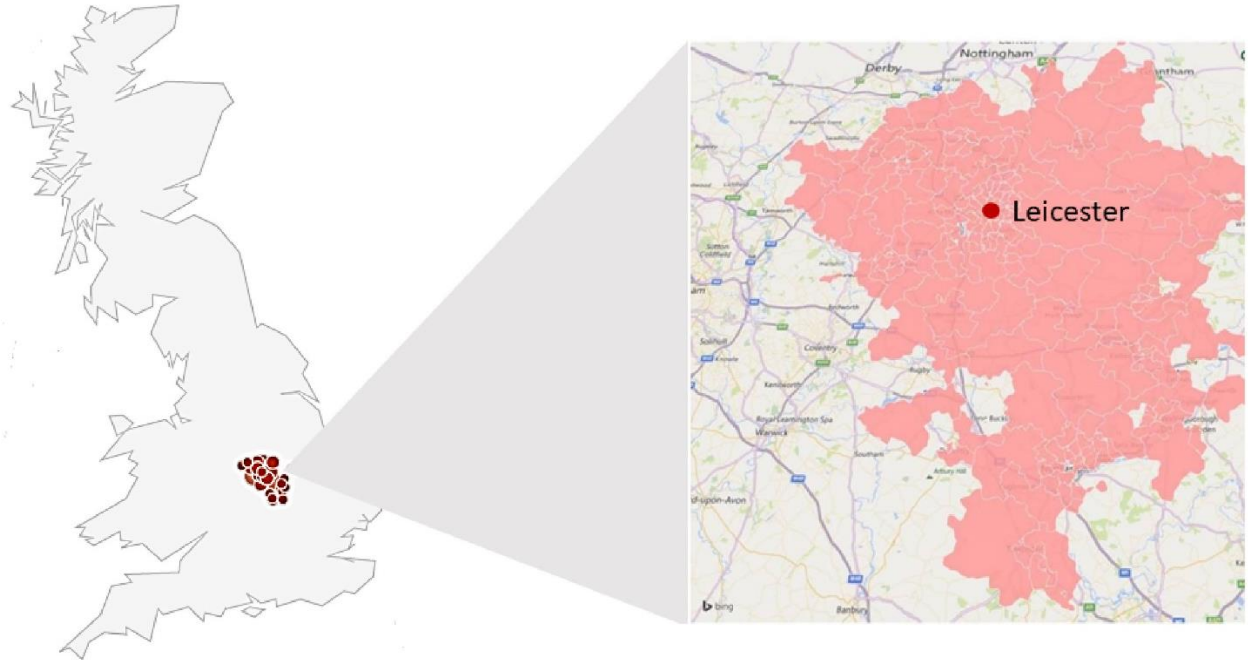

er review only

For peer review only - http://bmjopen.bmj.com/site/about/guidelines.xhtml

STROBE 2007 (v4) Statement—Checklist of items that should be included in reports of *cohort studies*

Section/Topic	Item #	Recommendation	Reported on page #
**Title and abstract**	1	(*a*) Indicate the study's design with a commonly used term in the title or the abstract	1,3
		(*b*) Provide in the abstract an informative and balanced summary of what was done and what was found	3
**Introduction**			
Background/rationale	2	Explain the scientific background and rationale for the investigation being reported	5
Objectives	3	State specific objectives, including any prespecified hypotheses	5
**Methods**			
Study design	4	Present key elements of study design early in the paper	5-9
Setting	5	Describe the setting, locations, and relevant dates, including periods of recruitment, exposure, follow-up, and data collection	5-9
Participants	6	(*a*) Give the eligibility criteria, and the sources and methods of selection of participants. Describe methods of follow-up	6
		(*b*) For matched studies, give matching criteria and number of exposed and unexposed	NA
Variables	7	Clearly define all outcomes, exposures, predictors, potential confounders, and effect modifiers. Give diagnostic criteria, if applicable	7-8
Data sources/ measurement	8*	For each variable of interest, give sources of data and details of methods of assessment (measurement). Describe comparability of assessment methods if there is more than one group	5-9
Bias	9	Describe any efforts to address potential sources of bias	7
Study size	10	Explain how the study size was arrived at	6
Quantitative variables	11	Explain how quantitative variables were handled in the analyses. If applicable, describe which groupings were chosen and why	8-9
Statistical methods	12	(*a*) Describe all statistical methods, including those used to control for confounding	8-9
		(*b*) Describe any methods used to examine subgroups and interactions	8-9
		(*c*) Explain how missing data were addressed	8-9
		(*d*) If applicable, explain how loss to follow-up was addressed	8-9
		(*e*) Describe any sensitivity analyses	8-9
**Results**			

For peer review only

Participants	13*	(a) Report numbers of individuals at each stage of study—eg numbers potentially eligible, examined for eligibility, confirmed eligible, included in the study, completing follow-up, and analysed	9-14
		(b) Give reasons for non-participation at each stage	9-14
		(c) Consider use of a flow diagram	
Descriptive data	14*	(a) Give characteristics of study participants (eg demographic, clinical, social) and information on exposures and potential confounders	9-10, supplementary materials
		(b) Indicate number of participants with missing data for each variable of interest	Table 1, Table 2, Table 3
		(c) Summarise follow-up time (eg, average and total amount)	11-12
Outcome data	15*	Report numbers of outcome events or summary measures over time	9-14
Main results	16	(*a*) Give unadjusted estimates and, if applicable, confounder-adjusted estimates and their precision (eg, 95% confidence interval). Make clear which confounders were adjusted for and why they were included	9-14, Figures
		(*b*) Report category boundaries when continuous variables were categorized	9-14, Tables 1-3
		(*c*) If relevant, consider translating estimates of relative risk into absolute risk for a meaningful time period	NA
Other analyses	17	Report other analyses done—eg analyses of subgroups and interactions, and sensitivity analyses	8
**Discussion**			
Key results	18	Summarise key results with reference to study objectives	14-16
**Limitations**			
Interpretation	20	Give a cautious overall interpretation of results considering objectives, limitations, multiplicity of analyses, results from similar studies, and other relevant evidence	14-16
Generalisability	21	Discuss the generalisability (external validity) of the study results	4- strengths and limitations
**Other information**			
Funding	22	Give the source of funding and the role of the funders for the present study and, if applicable, for the original study on which the present article is based	17

*Give information separately for cases and controls in case-control studies and, if applicable, for exposed and unexposed groups in cohort and cross-sectional studies.

Note: An Explanation and Elaboration article discusses each checklist item and gives methodological background and published examples of transparent reporting. The STROBE checklist is best used in conjunction with this article (freely available on the Web sites of PLoS Medicine at http://www.plosmedicine.org/, Annals of Internal Medicine at http://www.annals.org/, and Epidemiology at http://www.epidem.com/). Information on the STROBE Initiative is available at www.strobe-statement.org.

For peer review only
